# Dietary patterns in the Avon Longitudinal Study of Parents and Children

**DOI:** 10.1093/nutrit/nuv055

**Published:** 2015-09-22

**Authors:** Pauline M. Emmett, Louise R. Jones, Kate Northstone

**Affiliations:** *P.M. Emmett* is with the Centre for Child and Adolescent Health, School of Social and Community Medicine, University of Bristol, Bristol, UK. *L.R. Jones* and *K. Northstone* are with the School of Social and Community Medicine, University of Bristol, Bristol, UK.

**Keywords:** ALSPAC, children, cluster analysis, dietary patterns, fruit and vegetables, obesity, principal components analysis, pregnancy, reduced rank regression

## Abstract

Publications from the Avon Longitudinal Study of Parents and Children that used empirically derived dietary patterns were reviewed. The relationships of dietary patterns with socioeconomic background and childhood development were examined. Diet was assessed using food frequency questionnaires and food records. Three statistical methods were used: principal components analysis, cluster analysis, and reduced rank regression. Throughout childhood, children and parents have similar dietary patterns. The “health-conscious” and “traditional” patterns were associated with high intakes of fruits and/or vegetables and better nutrient profiles than the “processed” patterns. There was evidence of tracking in childhood diet, with the “health-conscious” patterns tracking most strongly, followed by the “processed” pattern. An “energy-dense, low-fiber, high-fat” dietary pattern was extracted using reduced rank regression; high scores on this pattern were associated with increasing adiposity. Maternal education was a strong determinant of pattern score or cluster membership; low educational attainment was associated with higher scores on processed, energy-dense patterns in both parents and children. The Avon Longitudinal Study of Parents and Children has provided unique insights into the value of empirically derived dietary patterns and has demonstrated that they are a useful tool in nutritional epidemiology.

## INTRODUCTION

The derivation of dietary patterns to describe diet, as opposed to investigating individual foods or nutrients, has become popular in nutritional epidemiology. Dietary patterns facilitate the study of the whole diet, recognizing that people consume foods in combination. They complement more traditional methods of examining diet–health relationships, which use individual foods or nutrients. Studies of dietary patterns and a particular disease outcome such as obesity have been largely limited to adults. The cross-sectional studies in adults often report conflicting relationships with disease.^1^ Prospective studies in adults have shown more consistent associations^2^; however, robust evidence from prospective studies for specific determinants of childhood development is limited.^3^

Most studies have used data-driven methods, such as principal components analysis (PCA) or cluster analysis, to derive dietary patterns.^4^^,^^5^ PCA utilizes correlations that exist between different food groups, identifying linear combinations of foods that are frequently consumed together. Each subject is given a score for every component/dietary pattern that emerges from the data. Cluster analysis assigns individuals into nonoverlapping groups (clusters) based on similarities between their dietary intakes.^6^ These groups are mutually exclusive. Some studies have directly compared patterns derived from these 2 methods and found some agreement between them, but in general they give different insights into the diets.^7^

Another method of deriving dietary patterns is reduced rank regression (RRR), which uses existing knowledge about disease etiology in combination with exploratory statistics to extract dietary patterns that are likely to be related to a specific disease.^8^^,^^9^ It is similar to PCA but has an extra step whereby intermediary (or response) variables known to be associated with an outcome of interest are used to inform the dietary patterns that are extracted. It requires existing evidence about factors associated with the disease under investigation that must be available to use in the analysis.

All 3 methods of obtaining dietary patterns described here were used in the Avon Longitudinal Study of Parents and Children (ALSPAC); the purpose of this narrative review is to summarize the findings of these analyses and compare and contrast the 3 methods within one cohort.

## LITERATURE AND STUDY METHODS

### Literature

All articles published online by the end of 2013 that derived dietary patterns based on ALSPAC data in children (from age 2 y) and their parents were reviewed. The 24 included articles are summarized in Table 1; 19 used PCA, 2 used cluster analysis, and 3 used RRR.^10–33^ The main dietary pattern method, the dietary data collection instrument, and the focus of each paper are described in Table 1. Relationships between dietary patterns during pregnancy and childhood outcomes are presented elsewhere.^34^ Dietary patterns have also been derived from the ALSPAC data obtained during the infancy of the cohort; these are described in a previous review.^35^

**Table 1:** Characteristics of the articles based on ALSPAC data included in the present review

Reference	Dietary pattern method	Diet assessment Method	Age or status of participant (child unless otherwise specified)	Focus of article
Northstone et al. (2007)^10^	PCA	FFQ	Adult females: pregnancy	DP, SEB
Northstone et al. (2008)^11^	PCA	FFQ	Adult females: pregnancy and 4 y postpartum	Change in DP
Northstone et al. (2010)^12^	PCA	FFQ	Adult males and females 4 y postpartum	DP, SEB, compared by sex, nutrients
North et al. (2000)^13^	PCA	FFQ	3 y	DP, SEB
Reilly et al. (2005)^14^	PCA	FFQ	3 y	Obesity at 7 y
Northstone et al. (2005)^15^	PCA	FFQ	4 and 7 y	DP, SEB
Northstone et al. (2008)^16^	PCA	FFQ	3, 4, 7, and 9 y	Stability of DP
Northstone et al. (2013)^17^	PCA	Dual FFQ	13 y	DP, SEB, nutrients
Northstone et al. (2012)^18^	PCA	Infant FFQ	2 y	DP, eating behavior
Northstone et al. (2008)^19^	PCA	FFQ	Females: pregnancy	DP, nutrients
Cribb et al. (2012)^20^	PCA	FFQ	3, 4, 7, and 9 y	DP, nutrients
Northstone et al. (2007)^21^	PCA	FFQ	Females: Pregnancy	DP adjusted for energy
Smith et al. (2013)^22^	PCA	FR	10 y	DP, input variables
Smith et al. (2014)^23^	PCA	FR	10 y	DP, adiposity
Smith et al. (2011)^24^	PCA, cluster	FR	7 y	DP comparison
Northstone et al. (2012)^25^	Cluster	FR	7, 10, and 13 y	DP longitudinally
Johnson et al. (2008)^26^	RRR	FR	5 and 7 y	DP, adiposity
Ambrosini et al. (2012)^27^	RRR	FR	7, 10, and 13 y	DP, adiposity
Ambrosini et al. (2014)^28^	RRR	FR	7, 10, and 13 y	DP, tracking
Feinstein et al. (2008)^29^	PCA	FFQ	3, 4, 7, and 9 y	School attainment
Northstone et al. (2012)^30^	PCA	FFQ	3, 4, 7, and 9 y	IQ at 8 y
Wiles et al. (2009)^31^	PCA	FFQ	4 y	Behavior, ADHD
Peacock et al. (2011)^32^	PCA	FFQ	7 y	Behavior
Easter et al. (2013)^33^	PCA	FFQ	3–9 y	DP in relation to maternal eating disorders

*Abbreviations*: ADHD, attention deficit hyperactivity disorder; Cluster, cluster analysis; DP, dietary pattern; FFQ, food frequency questionnaire; FR, food record; IQ, intelligence quotient; PCA, principal components analysis; RRR, reduced rank regression; SEB, socioeconomic background.

### Subjects

The ALSPAC is a birth cohort study that recruited pregnant women resident in 3 health districts of the county of Avon, around Bristol, in southwest England with an expected delivery date between April 1991 and December 1992 (n = 14 541 pregnancies).^36^^,^^37^ It was set up to investigate the ways in which genes and the environment, including diet, interact to affect the health, behavior, and development of children. The recruitment area covered the city of Bristol and surrounding towns and villages, including both urban and rural areas, with some industrial areas and some farming communities. Ethical approval for the study was obtained and the study was monitored throughout by the ALSPAC Law and Ethics Committee and the local research ethics committees. The study website contains details of all the data that is available through a fully searchable data dictionary.^38^

The cohort was population based and broadly representative, at recruitment, of the population of women with children aged <1 year in Avon.^36^ The children (n = 14 062 at birth; n = 13 988 alive at 1 year) have been followed by parental completion questionnaires, educational records, and hands-on assessment at dedicated research clinics.^36^ The indicators of socioeconomic background (SEB) of the families at recruitment are shown in Table 2. These include highest maternal educational attainment, age and housing tenure, ethnicity, smoking status, and prepregnancy body mass index (BMI) of the mothers. When the children were aged 7 years, they were invited to attend annual research clinics where hands-on measurements and tests were undertaken. At this time, an attempt was made to bolster the initial sample with eligible subjects who failed to join the study originally; this resulted in 706 additional families recruited. Therefore, after the age of 7 years the cohort consisted of 14 775 live births with 14 701 children alive at 1 year. Table 2 also shows the SEB of the mothers who completed the food frequency questionnaire (FFQ) for their child at ages 3 and 13 years and that of mothers of children who attended the research clinic and kept food records at age 7 years in comparison with the originally recruited mothers. Table 2 shows that the retained mothers have higher educational attainment, are older, and have more favorable health indicators than mothers whose children did not complete these parts of the study (for all differences *P* < 0.001).

**Table 2:** Socioeconomic background of the women recruited to the ALSPAC

Characteristic	Recruited mothers (n = 14 541)	Mothers who completed FFQ about the child at age 3 years (n = 10 137)		Mothers who completed food record for child at age 7 years (n = 7285)		Mothers who completed FFQ about the child at age 13 years (n = 6173)	
	n (%)	n (%)	*P*-value^a^	n (%)	*P*-value^b^	n (%)	*P*-value^c^
Maternal educational attainment			<0.001		<0.001		<0.001
No academic qualifications at age 16 y	3709 (25.5)	2519 (24.9)		1390 (19.1)		1167 (18.9)	
Academic qualification at age 16 y	4273 (29.4)	3479 (34.3)		2384 (32.7)		1989 (32.2)	
Education beyond age 16 y	4358 (30.0)	3786 (37.3)		2907 (39.9)		2607 (42.3)	
Missing	2201 (15.1)	353 (3.5)		604 (8.3)		410 (6.6)	
Maternal age at birth (years)			<0.001		<0.001		<0.001
<20	655 (4.5)	284 (2.8)		112 (1.5)		111 (1.8)	
20–24	2682 (18.4)	1577 (15.6)		911 (12.5)		732 (11.9)	
25–29	5369 (36.9)	4039 (39.8)		2718 (37.3)		2281 (36.9)	
30–34	3808 (26.2)	3130 (30.9)		2299 (31.5)		2040 (33.0)	
≥35	1382 (9.5)	1107 (10.9)		862 (11.9)		744 (12.1)	
Missing	645 (4.5)	0		383 (5.3)		265 (4.3)	
Housing tenure			<0.001		<0.001		<0.001
Mortgaged or owned	9757 (67.1)	7775 (76.7)		5641 (77.4)		4850 (78.5)	
Council and housing association rented	2138 (14.7)	1177 (11.6)		584 (8.0)		464 (7.5)	
Privately rented or other	1440 (9.9)	882 (8.7)		492 (6.8)		448 (7.3)	
Missing	1206 (8.3)	303 (3.0)		568 (7.8)		411 (6.7)	
Ethnicity			<0.001		<0.001		<0.001
White	11 927 (82.0)	9555 (94.3)		6549 (89.9)		5654 (91.6)	
Other ethnic groups	321 (2.2)	194 (2.0)		112 (1.5)		95 (1.5)	
Missing	2293 (15.8)	388 (3.7)		624 (8.6)		424 (6.9)	
Smoked in last trimester of pregnancy			<0.001		<0.001		<0.001
Yes	2413 (16.6)	1644 (16.3)		897 (12.3)		721 (11.7)	
No	8859 (60.9)	7343 (72.4)		5254 (72.1)		4593 (74.4)	
Missing	3269 (22.5)	1150 (11.3)		1134 (15.6)		859 (13.9)	
Maternal prepregnancy BMI			<0.001		<0.001		<0.001
<18.5	567 (4.0)	426 (4.2)		259 (3.6)		259 (4.2)	
18.5–24.99	8564 (58.9)	6817 (67.2)		4761 (65.4)		4162 (67.4)	
25–29.99	1737 (12.0)	1388 (13.7)		924 (12.7)		763 (12.4)	
≥30	631 (4.3)	491 (4.8)		317 (4.4)		264 (4.3)	
Missing	3033 (20.8)	1015 (10.0)		1024 (14.1)		725 (11.7)	

^a^ t-test comparing women in the original cohort (n = 14 541) with those who completed the food frequency questionnaire for their child at age 3 years (n = 10 137)

^b^ t-test comparing women in the original cohort (n = 14 541) with those who completed the food record for their child at age 7 years (n = 7285)

^c^ t-test comparing women in the original cohort (n = 14 541) with those who completed the food frequency questionnaire for their child aged 13 years (n = 6173)

*Abbreviations:* BMI, body mass index; FFQ, food frequency questionnaire.

### Food frequency questionnaire

Maternal diet was assessed by an unquantified FFQ sent to the mothers at 32 weeks gestation.^39^ Fathers were not contacted directly by ALSPAC. However, mothers were sent relevant paternal questionnaires to be passed on to their partners if they wished; this led to lower completion rates for paternal questionnaires. The pregnancy FFQ was adapted to cover the child’s diet at age 3 years and further adapted when repeated in both parents and the child 4 years after the birth of the study child.^12^ At this point, some minor modifications to the questions were made as a result of the experience gained from studying this cohort over time. For example, questions about breaded/coated poultry and fish and vegetarian pies were added because these were commonly eaten. Parentally completed questionnaires covering the child’s diet were obtained when the child was at the ages 3, 4, 7, 9, and 13 years. The last parentally completed questionnaire asked only about foods provided by the parents to their 13-year-old children; the adolescents themselves were asked to complete a questionnaire about foods eaten outside the home, such as school meals, take-away foods, and canned drinks.^17^ This was to ensure that information about the diet was collected from the perspective of both the parent and the child and that the child was not overloaded with questions. Copies of all the questionnaires used are available online.^40^

For the majority of foods/drinks, the person was asked to indicate how often the food was consumed currently using the following options: 1) never or rarely; 2) once in 2 weeks; 3) 1–3 times a week; 4) 4–7 times a week; and 5) more than once a day. For foods/drinks normally consumed daily, there were more detailed questions. These included recording how many cups of tea or coffee and the number of slices of bread consumed each day; the usual type of milk (full fat or other) consumed; the usual type of bread (white or brown/wholemeal) consumed; and the usual type of spread (butter or type of margarine or other) used. To apply quantitative meaning to the frequency categories, the data were numerically transformed into times consumed per week as follows: 1) 0; 2) 0.5; 3) 2; 4) 5.5; and 5) 10 times per week. Consumption of tea, coffee, bread, milk, and fat spreads were recorded on a daily rather than a weekly basis. Excluded from the dietary patterns analysis were participants whose FFQ had more than 10 dietary items unanswered. If 10 or fewer items were missing, the assumption was made that the person did not consume the missing items, and each was given a value of 0. No questions were asked about portion sizes; therefore, standard portion sizes^41^ tailored to the age of the person being assessed were used for the nutrient estimations. Nutrient intakes were calculated based on the frequency with which each food was consumed and the nutrient content of a portion of that food.^39^ A simpler version of the FFQ was administered when the children were aged 2 years^18^; this covered fewer foods than the later FFQs, and although dietary patterns were derived, it was not adequate to assess nutrient intakes.

### Food records

Prior to attending the research clinics, the parents (of 7-year-old children) or the child with parental help (at ages 10 and 13 y) was invited, by post, to record in a structured diary all foods and drinks consumed by the child over 3 individual days: preferably 1 weekend day and 2 weekdays.^42^^,^^43^ The participants could choose which days to record, so these were not necessarily consecutive. They were asked to bring the completed diaries to a research clinic, where, at ages 10 and 13 years, the child and accompanying parent were interviewed by a trained nutrition fieldworker who talked through the food record to clarify any uncertainties such as cooking methods and possible missing items. Foods and drinks consumed were recorded in household measures. The food records were transformed into weights and codes linkable to the energy and nutrient content corresponding to each of the foods eaten using DIDO software, developed by the Human Nutrition Research Unit in Cambridge, United Kingdom.^44^ When foods were not adequately described, weights were based on portion sizes for similar-aged children from national weighed dietary survey data.^45^ Weights were also obtained from manufacturers’ information and weights given on packets. Composite foods and recipes that did not have an equivalent in the food tables were broken down into their component parts. The data bank used for the nutrient analysis of the diet records included the 5th edition of McCance and Widdowson’s food tables^46^ and the supplements to the tables^47–55^ and were updated and expanded to cover all foods consumed. These data were used to generate average daily nutrient intakes and amount consumed of various groups of food/drink. Intakes from dietary supplements were not included in the nutrient calculations. Food records were also collected when the children were aged 5 years using the same methods in a 10% subsample of children in ALSPAC known as Children in Focus.^26^

### Misreporting of dietary intake

For the food record data, misreporting of energy intake (EI) was assessed at each age by an individualized method^56^ that calculated the ratio of reported EI to estimated energy requirement (EER) (EI:EER). Individual EERs were calculated using equations from an expert consultation report on human energy requirements, which allow for body weight, sex, and moderate physical activity.^57^ A 95% confidence interval (CI) for the accuracy of EI:EER was calculated by taking into account the amount of variation inherent in the methods used to estimate EI and EER.^58^ The CI for EI: EER, calculated for the dataset at age 7 years, for example, was 0.79–1.21. Therefore, reports of EI between 79% and 121% of EER were considered to be within the range of normal measurement error associated with estimating EI and EER and were defined as plausible reports. Under-reporting was defined as intakes below the cut-off and over-reporting as those above the cut-off. Slightly different cut-offs based on the data were applied at other ages. The amount of under-reporting increased with age (16% at 7 y; 61% at 13 y).

### Energy adjustment

Energy adjustment is necessary because energy is highly correlated with most other nutrients and related to body size^59^; thus, it may obscure underlying relationships with nutrients. The nutrient density of an individual food is the ratio of its nutrient content to total energy content. Nutrient-dense foods provide substantial amounts of micronutrients and energy content that is relatively low; thus, an overall diet that has a high micronutrient content after energy adjustment is more nutrient dense and has a better nutrient profile.

### Derivation of food groups

In the PCA and cluster dietary patterns analysis using food record data, all foods and drinks were grouped based on the questions asked in the FFQ.^15^ From these data, 95 food/drink groups were obtained; 14 were groups that had not been covered by specific questions in the FFQ but were eaten regularly and, therefore, were important to capture (these include batter and pastry products and sweet spreads). The 95 groups were reduced to 62 by combining some very similar groups (e.g., “citrus fruit” was combined with “other fruit” to form “fresh fruit”), and some groups were removed from the analysis because they were eaten by very few children and were unlikely to contribute to the dietary patterns (e.g., sugar-free confectionery and herbs and spices). For the RRR work, foods were grouped into 46 food groups according to differences in energy density and fat and fiber contents. For example, “fresh fruit” included canned fruit with no added sugar, which has low energy density, whereas “other fruit” included stewed and canned fruit with added sugar (added energy content) and dried fruit, which is energy dense due to its low water content.^26^

### Dietary patterns derived by principal components analysis

To perform PCA, all variables were standardized by subtracting the mean and dividing by the standard deviation for each variable. For the FFQ, the variables were the standardized average frequencies at which each food/drink was consumed per week; for food records, several input variables were tested. PCA forms linear combinations of the original observed variables by grouping together correlated variables, thus reducing the size of the dataset by identifying underlying components or patterns within the data.^60^ The coefficients defining these linear combinations are called factor loadings and represent the correlations of each food item with that component. The number of components (dietary patterns) that best represent the data is chosen on the basis of the scree plot,^61^ the size of the factor loadings, and the interpretability of the resulting patterns. Varimax rotation^60^^,^^62^ is applied; this redistributes the explained variance for the individual components and provides a simpler structure, increasing the number of larger and smaller loadings. A component score was created for each person for each of the dietary patterns identified; this was calculated by multiplying the factor loadings by the corresponding standardized value for each food and summing across the food items for each pattern. Each score has a mean of 0, and a high positive score suggests that the dietary pattern well describes the type of diet consumed by that person. Each time a dietary pattern score was derived, the PCA was repeated in 2 randomly selected halves of the dataset to assess the repeatability of the method. In all cases, the results were highly comparable for both the factor loadings and the component scores obtained, justifying the use of the whole dataset in the main analyses. Foods with loadings >0.3 on a component were considered to have a strong association with that component and were deemed to be the most informative in describing that particular dietary pattern. These foods were used to inform the labels assigned to each dietary pattern. The labels are rarely perfect in describing a dietary pattern but are useful in facilitating the reporting of the results. It should be noted that the labels do not represent exactly the same pattern in each group of people or at each time point so are not necessarily interchangeable.

### Dietary patterns derived from cluster analysis

Cluster analysis groups individuals into nonoverlapping clusters.^5^ Most cluster analysis algorithms do this based on measures of dissimilarity between pairs of subjects. The ALSPAC used the k-means method,^5^ whereby subjects are partitioned into clusters that minimize the sum of squares of distances from each subject to the cluster mean. The algorithm was run with 100 different starting clusters and the solution with the smallest sum of squares was chosen. The input variables for the cluster analysis was standardized by subtracting the mean and dividing by the range of the variables.^63^ For the FFQ, the input variables were standardized average weekly frequencies of intake of 57 food/drinks groups, whereas for the food records, the input variables were standardized average weights (g/d) consumed for each of 62 food/drinks groups. The reliability of the cluster solutions was tested by randomly splitting the data into halves and performing separate analyses on each half. This procedure was repeated 5 times, and the number of children allocated to each cluster was recorded. The least number allocated to different clusters indicated the most reliable solution. Analyses were run for 2–8 clusters for the FFQ data and for 2–6 clusters for the food record data. The best cluster solution in each case was chosen based on the amount of variation in the sample explained by the clusters, the size and ease of interpretation of the clusters, and reliability of the solution. The labels for the clusters were chosen to reflect the foods consumed in the highest frequency/amount on average in that cluster.

### Dietary patterns derived from reduced rank regression

RRR derives patterns that maximize the variation explained in a set of response variables, which are believed to be related to the outcome of interest. In this work, dietary energy density, fiber density, and percentage of EI from fat were selected as intermediate response variables based on evidence of a relationship with obesity, primarily in adults.^64–67^ Dietary energy density was calculated, excluding drinks, by dividing total food energy (in kJ) by total food weight (in g).^68^ Percentage of EI from fat was calculated by dividing energy (kJ) from fat by total EI (in kJ) and then multiplying by 100. Fiber density (in g/MJ) was calculated by dividing fiber intake (in g) by total EI (in MJ). Using SAS (version 9; SAS Institute, Cary, NC, USA) and the PROC PLS with RRR method option, 3 patterns were extracted in accordance with the number of response variables included.^26^^,^^27^ The pattern that explained the largest amount of variation in the response variables was used in further analyses. RRR was run in the whole sample and separately in boys and girls; the patterns produced had similar factor loadings to those derived from the whole sample. Therefore, factor loadings from the whole sample were used in the analyses. Pattern scores were calculated for each child as a linear combination of all food group intakes in the first pattern extracted.

### Socioeconomic background

Table 2 shows the SEB distributions at several time points in the study. Maternal educational attainment was assessed using the questionnaire sent out at 32 weeks gestation. Information was obtained on all of the qualifications of the mother; from this information a 5-point scale of highest educational attainment achieved was created using the following categories: no academic qualifications; vocational training (hairdressing, catering, etc.); O-level or equivalent examinations usually taken at age 16 years; A-level or equivalent examination usually taken at age 18 years; university degree.^36^ In some analyses, these categories have been further contracted to 3 categories: low, no academic qualification or vocational training; medium, O-level qualifications; high, A-level or degree-level qualifications. Maternal smoking status was assessed by questionnaires sent during pregnancy and infancy. Maternal age at delivery in years was calculated by subtracting the mother’s date of birth from the child’s date of birth. Housing tenure data were also collected during pregnancy and were categorized as follows: owned or mortgaged; public rented (council or housing association); private rented; and other. Questions on financial difficulties, including difficulty affording food, were derived from questionnaires during pregnancy and the child’s infancy and categorized as follows: no difficulty; some difficulty; great difficulty. Mothers were asked to report their prepregnancy weight and height by questionnaire during pregnancy; these values were used to calculate maternal prepregnancy BMI. The numbers of older and younger siblings in the family were obtained by questionnaire annually.

### Psychiatric assessment in mothers

At 12 weeks gestation, women were asked whether they had ever had any psychiatric problems, including eating disorders such as anorexia nervosa or bulimia nervosa.^69^

### Child’s intelligence quotient, school attainment, and behavior

Intelligence quotient (IQ) was measured using a validated, age-adjusted, shortened version of the Wechsler Intelligence scale for children at age 8 years.^70^ In the United Kingdom, there are standards regarding what children should be taught as part of the curriculum at each key stage.^29^ Children take national curriculum tests at the end of key stage 1 (aged 6–7 y) and key stage 2 (aged 10–11 y). Key stage scores obtained from the relevant education authorities in the National Pupil Database were matched to the ALSPAC data. For some children, the assessment administered by schools on entry and set by local education authorities when the children were aged 4–5 years were also available.

Childhood behavioral problems were measured using the strengths and difficulties questionnaire (SDQ).^71^ This questionnaire was completed by the parents at several ages throughout childhood. The SDQ is split into 5 subscales: hyperactivity, emotional symptoms, conduct problems, peer problems, and prosocial behavior (reverse scored).

### Child’s eating behaviors

Parents were asked annually by questionnaire about any difficulty they were experiencing feeding their child. The questions covered refusal of food, not eating enough, being choosy about food, and general difficulties with feeding. The answers were the parent’s subjective opinion and were not validated. There were also questions about whether the child was vegetarian or on a special diet of any type.

### Anthropometric and physical activity measurements

At each research clinic, height was measured to an accuracy of 0.1 cm using a Harpenden stadiometer (Holtain Ltd, Crymmych, Pembs, UK), and weight was measured to 0.1 kg using the Tanita Body Fat Analyser weighing scale (Tanita, West Drayton, Middlesex, UK). At ages 9, 11, 13, and 15 years, the children underwent a whole-body DXA (dual X-ray absorptiometry) scan (Lunar Prodigy DXA scanner; GE medical systems, Madison, WI, USA). The scan provided estimates of total fat mass, lean body mass, and bone mass. Fat mass index (FMI) was calculated by dividing fat mass (in kg) by height (in m^x^) to adjust for body size.^72^ In order to fully account for the correlation between fat mass and height an optimal power (x) by which height should be raise to create FMI was derived from the data ^26^^,^^72^; this power varied according to the age and sex of the child (e.g., x = 5.3 or 4.2 at age 11 years for boys and girls, respectively). There is no accepted cut-off to define excess adiposity with the use of fat mass, percentage of body fat, or FMI. However, when age- and sex-specific BMI cut-offs from the International Obesity Task Force^73^ were used to assess the occurrence of overweight in subjects, a 20% prevalence of overweight was found. For comparability, it was assumed that an equivalent percentage of children could be defined as having excess adiposity; therefore, those children in the top quintile of log FMI were categorized in this way. During the clinic visits, at ages 9, 11, 13, and 15 years, children were asked to wear an Actigraph accelerometer (Manufacturing Technology Incorporated, Fort Walton Beach, Florida) for a period of up to 7 days after the clinic visit.^74^ Data extracted from the accelerometers, expressed as the mean counts per minute during the period of wear, provided an objective measure of the physical activity of the child at each age.

### Statistical methods

Because this review includes many published articles, it is not practical to list all of the statistical methods used in each. However, descriptive statistics are provided within most of the articles. CIs are shown at the 95% level in all cases. Associations between variables were tested for using various statistical tests: paired *t*-tests, analysis of variance, and chi-square tests. Regression analyses were carried out after adjustment for major confounders, which depended on the outcome of interest and are described in the text for each study. The main confounding variables used in the various analyses were the indicators of SEB listed in Table 2. The specific methods for extracting dietary patterns are described above.

## RESULTS AND COMMENTARY

### Maternal and paternal dietary patterns from food frequency questionnaires using principal components analysis

Dietary patterns using PCA were obtained in the women at 32 weeks gestation during the study pregnancy and again when the study child was 4 years of age. During pregnancy, 5 patterns were obtained, explaining 31.3% of the variation in the data (n = 12 053).^10^ Table 3 shows the foods with factor loadings >0.3 on each pattern, the labels applied to the patterns, and the amount of the variance explained by each pattern. The “health-conscious” pattern had the strongest associations with the mother’s SEB; higher educational attainment, older age, owner occupancy, not smoking, and being a vegetarian were all associated with higher average scores on this pattern (all *P < *0.0001), and being overweight before pregnancy was associated with a lower average score (*P < *0.0001).^10^ The associations of SEB with the “processed” pattern were opposite those of SEB with the health-conscious pattern, except for maternal prepregnancy overweight, which was unrelated. The “confectionery” pattern was associated with younger maternal age, living in rented accommodation, and not being overweight prepregnancy (all *P < *0.0001). The “traditional” pattern showed very little association with SEB. The mother reporting herself to be a vegetarian was associated with a high average score on the “vegetarian” pattern (*P < *0.0001).

**Table 3:** Foods with high factor loadings on dietary patterns by principal components analysis in adults in the ALSPAC, including women in pregnancy (F1), women at 4 years after the birth of the study child (F4), and men at 4 years after the birth of the study child (M4)

PCA pattern	Foods with factor loadings >0.3^a^^,^^b^^,^^c^	Person: timing (variance explained)^a^^,^^b^^,^^c^
Health-conscious	Positive: brown/wholemeal bread, whole grain breakfast cereals, fish, cheese, pulses, pasta, rice, salad, fresh fruit, fruit juice F4 and M4: + yogurts F4 only: + plain potatoes, cooked vegetables M4 only: + water Negative: white bread M4 only: + meat pies, coated poultry, sausages, chips	F1: pregnancy (10.6%) F4: 4 years later (9.0%) M4: 4 years later (9.3%)
Traditional	Positive: all types of cooked vegetables and plain potatoes M4 only: + poultry, red meat, roast potatoes	F1: pregnancy (8.2%) M4: 4 years later (8.0%)
Processed	Positive: white bread, meat pies, sausages/burgers, fried foods, pizza, eggs, chips, roast potatoes, baked beans F4 only: + coated chicken and fish, peas Negative: brown/wholemeal bread	F1: pregnancy (4.9%) F4: 4 years later (7.5%)
Confectionery	Positive: biscuits, puddings, cakes/buns, sweets, chocolates, chocolate bars, crisps	F1: pregnancy (4.0%) F4: 4 years later (4.8%)
Processed/confectionery	Positive: biscuits, puddings, ice cream, cakes/buns, cold meats, coated poultry, meat pies, sausages, pizza, chips, baked beans, sweets, chocolates, crisps, fizzy drinks	M4: 4 years later (4.6%)
Vegetarian	Positive: meat substitutes, pulses, nuts F1 only: + herbal tea F4 only: + vegetarian pies Negative: poultry, red meat F4 only: + cold meats	F1: pregnancy (3.6%) F4: 4 years later (3.8%)
Semi-vegetarian	Positive: pizza, fish, vegetarian pies, meat substitutes, pulses, nuts, baked beans	M4: 4 years later (4.0%)

^a^ F1: Pregnancy data from Northstone et al. (2007)^10^

^b^ F4: Women at 4 years after the birth of the study child; data from Northstone et al. (2008)^11^

^c^ M4: Men at 4 years after the birth of the study child; data from Northstone et al. (2010)^12^

When the FFQ was repeated 4 years later, some minor changes to the questions (see Methods section) had been made, which changed the foods associated with the patterns slightly (see Table 3).^11^ Four distinct patterns explaining 25.1% of the variation in the data were extracted. When the pattern scores were divided into quintiles and compared between pregnancy and 4 years postpartum, approximately 40% of those in the top or bottom quintile for the health-conscious and processed patterns during pregnancy remained in that same quintile for that pattern. The weighted kappa for each of the 4 consistent patterns was between 0.31 and 0.27, showing only a moderate amount of agreement between the dietary patterns at the 2 time points. The mean score on the health-conscious pattern decreased, whereas that on the processed pattern increased (both *P < *0.0001), suggesting that women were moving toward a more processed dietary pattern. An applied analysis was performed that used the factor loadings obtained from the PCA during pregnancy to calculate the PCA scores on the data obtained 4 years later (to demonstrate any real changes in the original pattern scores).^11^ When the stability of the diet was reassessed by dividing the applied scores into quintiles and comparing them to the pregnancy quintiles, the health-conscious pattern scores were relatively stable; however, only 20% of the processed pattern scores remained in the original extreme quintiles. These differences in patterns observed between pregnancy and 4 years postpartum may have been influenced by the presence of children in the household; 46% of the women were first-time mothers at the arrival of the study child so were less likely to have had children in the household during this pregnancy.

At the same time that the women completed the FFQ at 4 years postpartum, they were asked to pass an identical FFQ to their partner to complete; this was used in a PCA to determine dietary patterns in men.^12^ Data were available for 4681 men, and 4668 had data from their female partners for comparison. Four dietary patterns explaining 25.9% of the variability of the data were extracted for the men; however, the patterns were slightly different from those found in the women (see Table 3). There were strong positive correlations between the matched men’s and women’s scores on the health-conscious (0.36; *P < *0.001) and vegetarian/semi-vegetarian patterns (0.38; *P < *0.001) and a strong negative correlation between the men’s scores on the health-conscious pattern and the women’s on the processed pattern (−0.48; *P < *0.001); other correlations were weaker.^12^

The men’s educational attainment was strongly positively associated with scores on the health-conscious pattern (*P < *0.0001), although not with scores on the other 3 patterns. Men’s age was positively associated with scores on the health-conscious pattern but negatively associated with scores on the processed/confectionery pattern. Higher frequency of smoking in men was strongly associated with a lower average scores on the health-conscious pattern. Men who identified themselves as vegetarian had a high average score on the semi-vegetarian pattern, a slightly lower average score on the health-conscious pattern, and low average scores on the other 2 patterns. These results suggest that men in this study who are mostly living within families have slightly different patterns of diet from their female partners.

### Dietary patterns from the food frequency questionnaires in childhood using principal components analysis

The first analysis performed in childhood was at age 3 years (n = 10 139)^13^; at the time of the ALSPAC analysis, no other published study had derived dietary patterns using PCA in childhood. Four dietary patterns were extracted that explain 23.5% of the variation in the data; this is a very similar amount to previous studies in adults. The foods with high factor loadings on the patterns are shown in Table 4. The most significant SEB variable in explaining differences in dietary pattern scores at age 3 years was maternal education; higher maternal educational attainment was associated with lower average “junk” scores but higher “healthy” and “snacks” scores (all *P < *0.0001).^13^ Other notable associations were as follows: boys were less likely than girls to have high scores on the healthy and “traditional” patterns (both *P < *0.0001); presence of older siblings was associated with higher average scores on the junk and snacks patterns and lower average scores on the traditional pattern (all *P < *0.0001); living in public rented housing was strongly positively associated with the junk pattern and negatively with the snacks pattern (both *P < *0.0001); having financial difficulty was also strongly positively associated with the junk pattern and negatively with the snacks pattern (both *P < *0.0001). All of these associations were independent of each other. These findings suggest that the types of food preschool children eat are influenced by the social environment of the family.

**Table 4:** Summary of factor loadings of foods in the dietary patterns extracted using principal components analysis from food frequency questionnaires at 3, 4, 7, and 9 years

Food item	Snacks^a^	Junk/processed^a^^,^^b^				Traditional^a^				Healthy/health-conscious/vegetarian^a^^,^^c^			
	3 y	3 y	4 y	7 y`	9 y	3 y	4 y	7 y	9 y	3 y	4 y	7 y	9 y
Variance explained	(4.0%)	(7.88%)	(7.31%)	(7.32%)	(7.2%)	(4.14%)	(6.48%)	(7.03%)	(7.9%)	(7.43%)	(3.85%)	(3.81%)	(4.1%)
White bread	−0.070	**0.344**	0.287	**0.302**	**0.308**	0.050	−0.001	0.023	−0.176	−0.232	−**0.425**	−**0.339**	−0.213
Nonwhite bread			−0.266	−0.290	−0.296		−0.019	−0.026	0.231		**0.489**	**0.418**	0.251
Biscuits	**0.531**	**0.318**	**0.496**	**0.509**	**0.522**	−0.031	0.076	−0.034	−0.081	−0.167	−0.098	−0.082	−0.014
Ice cream			**0.510**	**0.510**	**0.496**		0.009	0.047	0.085		0.096	0.086	0.064
Milk-based puddings and custard			**0.311**	**0.367**	**0.414**		0.278	0.230	**0.378**		0.141	0.246	0.138
Other puddings^d^	**0.552**	−0.032	0.267	0.289	**0.332**	0.151	0.262	0.240	**0.368**	0.097	0.175	0.255	0.139
Cakes/buns	**0.509**	0.211	**0.356**	**0.346**	**0.327**	−0.012	−0.076	0.032	0.152	0.016	0.103	0.144	0.078
Poultry	0.136	0.011	0.054	−0.099	0.058	**0.603**	**0.572**	**0.627**	**0.457**	−0.009	−0.115	−0.154	−**0.480**
Red meat	0.118	0.036	0.059	0.102	0.071	**0.619**	**0.606**	**0.608**	**0.394**	−0.087	−0.269	−0.075	−**0.539**
Cold meats			0.151	0.172	0.164		0.190	0.239	0.149		−0.159	−0.187	−**0.349**
Coated poultry products			**0.356**	**0.387**	**0.406**		0.097	0.212	−0.012		−0.151	−0.207	−0.296
Meat pies			0.274	**0.325**	**0.326**		0.113	0.131	0.055		−0.097	−0.075	−0.133
Sausages, burgers	−0.125	**0.468**	**0.356**	**0.395**	**0.361**	0.066	−0.028	−0.009	−0.076	0.124	−0.153	−0.161	−0.173
Pizza	0.078	**0.321**	**0.306**	**0.301**	**0.315**	−0.086	−0.061	−0.048	0.051	0.244	0.203	0.234	0.147
Fish^e^	0.061	0.076	−0.004	−0.071	−0.025	0.193	**0.310**	**0.312**	**0.427**	**0.301**	**0.340**	**0.319**	0.083
Eggs	−0.029	0.164	0.147	0.126	0.089	0.159	0.172	0.171	**0.323**	**0.322**	0.266	0.299	0.101
Cheese	**0.314**	−0.098	0.084	0.052	0.070	0.039	0.092	0.085	0.243	**0.319**	**0.336**	**0.320**	0.225
Vegetarian pies			0.056	0.095	0.122		−0.043	−0.114	0.137		**0.410**	**0.448**	**0.448**
Meat substitutes (soy, tofu, etc.)	−0.046	0.015	−0.056	−0.051	−0.022	−0.280	−0.128	0.208	0.102	**0.529**	**0.524**	**0.523**	**0.600**
Pulses	−0.064	−0.019	−0.098	−0.110	−0.100	−0.074	0.079	−0.010	0.233	**0.588**	**0.485**	**0.486**	**0.495**
Nuts			−0.037	−0.049	−0.005	−0.280	−0.129	−0.089	0.157		**0.408**	**0.401**	**0.411**
Chips	0.123	**0.484**	**0.479**	**0.511**	**0.501**	−0.047	−0.090	−0.050	−0.120	−0.085	−0.074	−0.072	−0.080
Roast potatoes		−	0.246	0.245	0.273		**0.381**	**0.409**	0.255		−0.210	−0.182	−**0.325**
Potatoes (not chips)	0.125	0.147	−0.017	0.009	0.054	**0.489**	**0.462**	**0.448**	**0.436**	−0.024	−0.015	0.075	−0.151
Pasta	0.174	−0.068	−0.072	−0.099	−0.102	0.068	0.198	0.197	**0.422**	**0.500**	**0.476**	**0.417**	0.150
Rice	−0.026	0.064	−0.044	−0.020	−0.053	0.197	0.268	**0.324**	**0.465**	**0.525**	**0.358**	**0.352**	0.064
Baked beans/canned pasta^f^	0.150	0.204	**0.356**	**0.377**	**0.414**	0.115	0.126	0.131	0.106	0.078	0.054	0.038	−0.065
Green vegetables	−0.094	0.059	−0.069	−0.105	−0.141	**0.610**	**0.735**	**0.702**	**0.634**	0.241	0.185	0.255	−0.039
Root vegetables	−0.004	−0.040	−0.074	−0.078	−0.077	**0.554**	**0.644**	**0.584**	**0.633**	0.278	.0.203	0.266	−0.014
Peas	0.099	0.006	0.026	−0.014	0.037	0.241	**0.568**	**0.517**	**0.440**	**0.447**	0.082	0.156	−0.049
Sweetcorn			−0.056	−0.028	−0.014		**0.398**	**0.412**	**0.464**		0.275	0.270	0.037
Salad	0.100	−0.048	−0.046	−0.115	−0.177	0.131	0.265	0.283	**0.452**	**0.445**	**0.434**	**0.421**	0.236
Fresh fruit	**0.385**	−0.226	0.046	−0.004	−0.030	0.167	0.278	0.296	**0.453**	**0.356**	**0.351**	**0.339**	0.114
Fruit juice	0.116	−0.198	0.020	0.041	−0.073	−0.044	0.045	0.061	0.215	**0.383**	**0.341**	0.251	0.177
Fizzy drinks	0.040	**0.582**	**0.450**	**0.484**	**0.451**	−0.017	−0.032	0.015	−0.081	−0.092	−0.146	−0.084	−0.069
Tea/coffee	−0.274	**0.411**	0.009	−0.060	−0.006	−0.185	0.029	0.035	0.004	−0.069	0.015	−0.002	0.001
Water	−0.068	−0.087	−0.073	−0.110	−0.177	0.065	0.124	0.135	0.295	**0.367**	0.281	**0.301**	0.170
Flavored milk drinks	−0.067	**0.301**	0.054	0.189	0.157	0.079	0.084	0.060	0.133	0.067	0.068	0.128	0.089
Sweets	0.139	**0.517**	**0.490**	**0.522**	**0.503**	−0.009	−0.003	-0.016	−0.054	−0.058	−0.077	−0.013	0.030
Chocolate	0.258	**0.515**	**0.486**	**0.516**	**0.520**	−0.019	−0.047	−0.041	−0.095	−0.102	−0.098	−0.062	0.004
Crisps	0.291	**0.456**	**0.465**	**0.439**	**0.426**	−0.019	−0.112	−0.068	−0.104	−0.145	−0.099	−0.076	−0.028

^a^ Patterns of similar type over the ages are grouped together. Boldface numbers indicate loadings >0.3.

^b^ This pattern has been labeled “junk” or “processed.”

^c^ This pattern was labeled “health-conscious/vegetarian” at 9 years.

^d^ At age 3 years, only 1 question was asked regarding puddings.

^e^ At age 3 years, breaded fish products were included in this group; they were treated separately at all other time points.

^f^ At age 3 years, canned pasta was not included in this group, as it was not asked about.

Modified from Northstone et al. (2008)^16^

The dietary patterns determined at age 3 years were used in analysis that looked at obesity at age 7 years defined by the 95^th^ percentile of BMI from the 1990 UK reference data.^14^ The initial positive relationship of the junk pattern with obesity was not robust to adjustment to other factors, such as maternal prepregnancy BMI.^14^

PCA was performed on the FFQ data collected when the children were aged 4 years (n = 9550) and 7 years (n = 8286).^15^ At both ages, 3 dietary patterns best described the diet (Table 4 shows the factor loadings for foods associated with each pattern) and explained 17.6% and 18.2% of the variation of the foods eaten at ages 4 years and 7 years, respectively. The patterns were very similar at the 2 ages and similar to the first 3 patterns found at age 3 years. The SEB factor most strongly associated with the patterns was, again, maternal education, and the relationships were identical to those found at age 3 years.^15^ Living in public rented housing and having financial difficulties showed smaller correlations at these ages than they had at age 3 years. Having older siblings was again associated with higher average scores on the processed pattern (*P < *0.0001 at both ages), as was having younger siblings at age 7 years (*P < *0.0001). Some childhood eating behaviors were assessed in this analysis: if the child was a vegetarian, they were likely to have lower average scores on the processed and traditional patterns and higher scores on the health-conscious pattern at both ages (all *P < *0.0001); if the child was considered to be a difficult eater, they had higher average scores on the processed pattern and lower average scores on the traditional and health-conscious patterns at both ages (all *P < *0.0001); boys had slightly lower average scores than girls on the traditional pattern (*P < *0.0001 at both ages). These analyses suggest that the characteristics of the child as well as the SEB of the family are important in determining the type of diet eaten by the child.

When PCA was performed at age 9 years (n = 8010)^16^ the traditional and processed patterns were similar to those at younger ages (Table 4). However, the health-conscious pattern differed by showing negative factor loadings on meat and poultry (Table 4); therefore, this pattern was labeled “health-conscious/vegetarian.”

In light of the similarity between dietary patterns obtained at these 4 ages between 3 years and 9 years, the stability of the patterns over time was assessed.^16^ The correlations between scores over time for each of the 3 main patterns (n = 6177 with data at all ages) were reasonable given the large sample size. Correlation coefficients between adjacent ages were larger (0.53–0.69; all *P < *0.0001) than when the youngest age was compared with the oldest age (0.35–0.46; all *P < *0.0001). The snacks pattern only appeared at age 3 years and was associated with the processed but not traditional or health-conscious patterns at later ages. Comparing the average scores for each pattern at each age, only the processed pattern score increased over time (*P < *0.0001). When the data were categorized into quintiles, the highest amount of agreement was between the ages of 4 years and 7 years (weighted kappa values of 0.42, 0.44, and 0.47 for the processed, traditional, and health-conscious patterns, respectively). In general, the amount of agreement was lower for the longer time gaps. Taking these results together shows a gradual evolution of the pattern of eating over childhood, suggesting that assessing diet at only one age during childhood may not be sufficient to characterize the diet.

This is further illustrated by the PCA that was performed on the FFQ collected when the children were adolescents (aged 13 y).^17^ This FFQ had been split into 2 questionnaires; one given to the parents asking about foods they provide and a second, shorter questionnaire given to the adolescents asking about foods they consumed outside of the home. When the 2 sets of data were combined, four dietary patterns explaining 20.8% of the variation were extracted. The first pattern had high positive loadings for meat, fish, eggs, cheese, rice, pasta, potatoes, vegetables, salad, fruit, pulses, brown/wholemeal bread, yogurt and dairy-based desserts, puddings, salad dressings, and water with high negative loadings for white bread and coated poultry products. It combined foods associated with both the health-conscious and traditional patterns at previous ages so was labeled “traditional/health-conscious”; this pattern explained 6.6% of the variance. The second pattern was similar to previous “processed” patterns; it explained 6.1% of the variance. The next pattern had high loadings on crisps, biscuits, chocolate, sweets, squash, and carbonated drinks and was labeled “snacks/sweet drinks”; it explained 4.2% of the variance. The final pattern had high positive loadings for vegetarian-style foods (such as meat substitutes, nuts, and pulses) and high negative loadings for meats and was, therefore, labeled “vegetarian”; it explained 3.8% of the variance. This was the first time a recognizably vegetarian-style of eating had been found among the dietary patterns of children, and it followed the semi-vegetarian pattern extracted for the 9-year-olds.

Pattern scores were higher, on average, for the traditional/health-conscious and vegetarian patterns for females and as maternal educational attainment increased.^17^ For both the processed and snacks/sweet drinks patterns, average scores were higher in males, if mothers were younger or less educated, and when 2 or more older or younger siblings were present. The characteristics of the child were, again, strongly associated with the dietary patterns. In particular, if the child was recorded as being vegetarian by their parents, their average score on the vegetarian pattern was very high; they also tended to have high average scores on the traditional/health-conscious pattern and low average scores on the other 2 patterns. Adolescents whom parents found difficult to feed (refused food, did not eat enough, or were choosy with food) tended to have lower average scores on the traditional/health-conscious pattern and higher average scores on the other 3 patterns. Compared with adolescents who ate a mixture of meal types, those who ate school dinners every day had higher average scores on the traditional/health-conscious and processed patterns and lower average scores on the snacks/sugared drinks pattern; those who ate packed lunches every day had the opposite associations with the dietary pattern scores. These data provide further evidence of the evolution of dietary patterns over time and suggest that the strongest influences on dietary patterns remain maternal educational attainment and child feeding behaviors. However, they also suggest that the type of food provision in schools can influence the pattern of eating.

To complete the series of dietary patterns from FFQs in childhood, a PCA was performed on the data collected when the children were aged 2 years.^18^ These data cover the period at the end of infancy and beginning of childhood, and the list of foods covered was shorter than that used at age 3 years and beyond. Three patterns explaining 25.5% of the variance were extracted. The first was labeled “family foods” (12.9% of variance) and was similar to the traditional pattern at later ages; the third was also similar to a pattern found at later ages and was labeled “health-conscious” (5.2% of variance). However, the middle pattern was different from later patterns, with 2 types of foods loading highly on to it: sweet foods, such as confectionery, carbonated drinks, and flavored milks, and easily prepared savory foods, such as crisps, potatoes, baked beans, peas, and soup. This pattern was labeled “sweet and easy”; it explained 7.4% of the variance in the data.

The relationships between SEB and the family foods and health-conscious dietary patterns were very similar to those for the equivalent patterns at later ages. High scores on the sweet and easy pattern were evident with lower maternal educational attainment, living in public rented housing, and presence of older siblings (all *P < *0.0001).^18^ Higher scores on this pattern were also associated with the family having increasing financial difficulties (*P = *0.007). Children who were “very choosy” about foods had lower average scores on all 3 patterns compared with those who “ate anything.” This was particularly true for the health-conscious pattern (fully adjusted average pattern score for “very choosy” children compared with the “ate anything” reference group: −0.324 [Standard Error: 0.04]; *P < *0.0001). These data covering the whole of childhood show that the relationship between dietary patterns and child and family characteristics are similar at each age.

### Dietary patterns from principal components analysis in relation to nutrient intakes estimated from food frequency questionnaires

The first published article from the ALSPAC relating dietary patterns to estimated nutrient intakes used the pregnancy diet and broke new ground in this area of research.^19^ It assessed linear correlations between dietary pattern scores and nutrient intakes, both with and without energy adjustment, as well as categorical associations between quintiles of dietary pattern scores and mean energy-adjusted nutrient intakes in each quintile; clear associations were found. In particular, the highest quintile of the processed pattern score was associated with higher EI (difference, 2.5; 95% CI, 2.4–2.6 MJ/d) with higher fat (difference, 7.9; 95% CI, 7.3–8.5 g/d) and lower total sugars intake (difference, −21.6; 95% CI, −23.2 to −20.0 g/d) than the lowest quintile. This was combined with lower intakes of calcium, magnesium, iron, vitamin C, folate, and most micronutrients (all *P < *0.0001). The highest quintile on the confectionery pattern score compared with the lowest was also associated with increased energy (difference, 3.1; 95% CI, 3.0–3.2 MJ/d) but with decreased protein (difference, −14.4; 95% CI, −15.1 to −13.8 g/d) and increased total sugars intake (difference, 32.7; 95% CI, 31.0–34.4 g/d), again combined with decreased intakes of most micronutrients (all *P < *0.0001). In contrast, the health-conscious and traditional patterns showed much smaller correlations with energy. There were lower fat (difference, ∼ −5 g/d) and higher protein intakes (difference, ≥10 g/d) in the highest compared with the lowest quintiles of both pattern scores, along with higher intakes of almost all micronutrients (*P < *0.0001). The vegetarian pattern score did not show consistent correlations with the micronutrients, but protein intake was lower in the highest than the lowest quintile of pattern score (difference, −14.1; 95% CI, −15.1 to −13.2 g/d; *P < *0.0001).

In the male partners of the ALSPAC mothers and in the study children at each age (3, 4, 7, 9, and 13 y), only linear correlations were calculated; there were many similarities with the associations shown in the women during pregnancy despite slight variations in the patterns found.^12^^,^^17^^,^^20^ Scores on the processed/confectionery types of patterns showed the strongest positive correlations with EI, and after energy adjustment, almost all nutrients, except total sugars and saturated fat, were negatively associated. High scores on these patterns, therefore, described energy-dense, nutrient-poor diets at each age. The scores on the health-conscious types of patterns showed the lowest positive correlations with EI and were negatively associated with fat intake. Energy adjustment strengthened the associations with protein, fiber, and almost all micronutrients, showing that high scores on these patterns described nutrient-rich diets. There were moderate correlations between scores on the traditional types of patterns and EI. Adjusting for energy reduced positive associations with protein, fiber, and micronutrients slightly, but on the whole, high scores on these dietary patterns described relatively nutrient-rich diets. This was less evident with high scores on the vegetarian/semi-vegetarian dietary patterns because associations with micronutrients were considerably reduced on energy adjustment.

Before energy adjustment, at least 50% of the variance of many nutrients was explained by the dietary patterns at each age; this was, for the most part, reduced after adjustment for EI.^12^^,^^17^^,^^19^^,^^20^ However, for protein, fiber, magnesium, zinc, folate, carotene, and niacin, more than 50% of the variance was still explained at most ages after energy adjustment. Many of these are key nutrients for growth (protein, zinc)^75^ and development (iron, folate),^76^ and some have been associated with cancer outcomes (fiber, folate).^77^ These results suggest that dietary patterns extracted using PCA are able to provide insight into the likely adequacy of whole diets as consumed.

In the analyses relating dietary patterns to nutrient intakes, the energy adjustment had been carried out after the dietary patterns had been extracted. There remained the question as to whether adjusting for EI prior to entering the foods into the PCA would change the results.^21^ Using the FFQ collected at 32 weeks gestation, the PCA was performed in 2 ways: the first with the standardized weekly frequencies of the foods consumed (as already described), the second with the standardized frequencies adjusted for EI using the residuals method.^59^ When the energy-adjusted foods were used in the PCA, only 4 dietary patterns were obtained instead of the original 5. The processed pattern was lost, and the foods loading highly on this pattern in the unadjusted data now had increased negative loadings on the health-conscious dietary pattern. The other patterns—traditional, confectionery, and vegetarian—had very similar factor loadings on the foods, regardless of whether or not they were energy adjusted. The amount of variance in the data that was explained by the patterns overall was slightly less (adjusted, 26.9%; unadjusted, 32.4%). The 4 remaining patterns were highly correlated between the adjusted and unadjusted versions, and the unadjusted processed pattern was negatively correlated with both the adjusted health-conscious and confectionery patterns. The adjusted pattern scores showed lower correlations with nutrient intakes and explained less of the variance in the data than the unadjusted pattern scores. This was due to a reduction in the variability in the data when energy adjustment was carried out.

The effect of the 2 methods of adjustment on associations between dietary patterns during pregnancy and birth weight were assessed: in both cases, an increase in the health-conscious pattern score was associated with an increase in birth weight (35–40 g increase in birth weight for a 1 standard deviation increase in health-conscious score, regardless of whether energy adjustment was made before or after the PCA). It should be noted that in this analysis many other factors that are known to influence birth weight were not considered.^21^ This work suggests that future studies should ideally adjust for energy after the PCA has been performed so that the true effects of energy adjustment can be assessed. Furthermore, some outcomes of interest may have no association with EI, and this may be masked by prior adjustment.^21^

### Dietary patterns from food records in childhood using principal components analysis

An analysis of the data from the food records collected at age 10 years (n = 7473) explored the methodological issue of whether the patterns obtained using PCA would be affected by the type of input variable used to describe the food groups.^22^ Four different input variables were used: weight (g/d consumed on average), energy-adjusted weight (using the residuals method), percentage contribution of each food group to EI, and binary (consumed/not consumed). In the first 3 analyses, input variables were standardized before entry in the PCA so that foods consumed in large quantities did not dominate the PCA; this was not appropriate for the binary variables.

The results showed very little difference between the patterns derived from the absolute weights and the energy-adjusted weights of the food groups.^22^ Three similar patterns explaining about 10% of the variation were extracted: brown/wholemeal bread, fruit, vegetables, fruit juice, and water loaded highly on the first pattern; meat, roast potatoes, vegetables, and puddings loaded on the second; white bread, crisps, biscuits, and diet soft drinks loaded on the third pattern. When the percentage of energy from the food groups was used, 12% of the variation was explained and a fourth pattern was derived to add to 3 that were quite similar to those above. This fourth pattern had high loadings on reduced-fat milk, breakfast cereal, and biscuits. The main differences in factor loadings were for drinks that provide minimal energy—e.g., water and diet drinks. The results suggest there is no advantage in energy adjustment being applied before the derivation of the dietary patterns (similar to the finding with the FFQ during pregnancy^21^) and that because using percentage of energy as the input variable eliminates low-energy drinks from the patterns, it leads to unnecessary loss of information.

The use of binary variables in the PCA gave slightly different patterns, which might represent dietary choices and may provide insight into where dietary change could be influenced. Four patterns explaining 17% of the variation were extracted.^22^ The first loaded on meat, roast potatoes, vegetables, and gravy. The second loaded positively on brown/wholemeal bread, fruit, cakes, chocolate, fruit juice, regular soft drinks, and water and negatively on diet drinks and roast potatoes. Loadings on the third were positive for white bread, chips, crisps, chocolate, sweets, cakes, biscuits, carbonated sweet drinks, and diet drinks. The fourth had positive loadings on breakfast cereals, reduced-fat milk, margarine, and diet soft drinks but negative loadings on full-fat milk, butter, and regular soft drinks. These possible insights into food choice suggest that further work using binary variables is justifiable.

The associations between body composition at ages 9 years and 11 years and the PCA dietary patterns obtained at age 10 years using the food group weights (g/d) were examined. The analyses explored the differences between boys and girls and between those who were under-reporters of EI and those who were plausible reporters.^23^ Three dietary patterns were identified: the first was labeled “health-aware” and had positive loadings on fruit and vegetables, brown/wholemeal bread, pasta, cheese, and fish and negative loadings on chips, crisps, processed meat, and carbonated drinks. The foods that loaded positively on the second pattern, which was labeled “traditional,” were meat, roast potatoes, vegetables, batter/pastry products, gravy, and puddings. For the third pattern, which was labeled “packed lunch,” the foods with the highest factor loadings were white bread, margarine, cheese, cold meats, savory spread, and diet squash. In this analysis, many of the foods often associated with a processed pattern were negatively loaded on the health-aware pattern.

There were differences in average standardized pattern scores between boys and girls and between plausible reporters and under-reporters.^23^ Girls had a higher average score than boys on the health-aware pattern. Under-reporters had lower average scores than plausible reporters for all of the patterns in girls and for the traditional and packed lunch patterns in the boys. Under-reporters of both sexes had higher fat mass and lean mass and were taller, on average, at the ages of 9 years and 11 years than plausible reporters, although they reported lower EIs. The only consistent association between the dietary pattern scores and body composition was in under-reporters, in whom, in both boys and girls, lower average fat mass was associated with higher scores on the health-aware pattern. There was also a trend in this direction among the girls who were plausible reporters, but in the boys, the associations were not robust to adjustment for maternal prepregnancy BMI, smoking during pregnancy, age, and educational attainment (at the time of pregnancy with the study child) or for physical activity (by accelerometer). These results suggest that diet is reported slightly differently between the sexes and by misreporting status when food records are used.

### Dietary patterns from food frequency questionnaires and food records in childhood using cluster analysis

Cluster analysis was used to obtain dietary patterns, and the results were compared with those from PCA in the children at age 7 years using the FFQ.^24^ The cluster analysis grouped children into mutually exclusive groups that were similar to each other in terms of foods consumed. Whether different cluster solutions were evident in boys and girls was assessed; very little difference was found between the sexes, so they were combined. The 3-cluster solution was chosen as being the most reliable (<0.5% allocated to different clusters), and it explained a reasonable amount of the variation (14.5%). The foods/drinks that were the main discriminators between the 3 clusters were milk (full-fat or low-fat), bread (brown/wholemeal or white), and, to a lesser extent, salad, carbonated drinks, pasta, biscuits, and chips. The “processed” cluster was the largest (n = 4177), with more frequent consumption of white bread, soft drinks, biscuits, chips, crisps, sweets, and ice cream than in the other 2 clusters. The 2 other clusters were of similar size (n = 2065 and n = 2037). In the “plant-based” cluster, children consumed brown/wholemeal bread, low-fat milk, fruit, fruit juice, vegetables, salad, meat substitutes, cheese, fish, pasta, rice, and wholegrain breakfast cereals more frequently than in the other clusters. In the “traditional British” cluster, children consumed more full-fat milk, meat, coated fish, roast potatoes, yogurt, milk puddings, cakes, low-fiber breakfast cereals, chocolate, tea/coffee, and flavored milk than in the other clusters. When comparing the clusters with dietary pattern scores obtained using PCA, the plant-based cluster had a strong positive association with the PCA health-conscious pattern score and a strong negative association with the PCA processed pattern score. The processed and traditional British clusters were much less strongly associated with the PCA scores.

The age and educational attainment of the mother were strongly independently associated with the child’s cluster membership.^24^ The younger the mother, the more likely the child was to be in the processed cluster. The more educated the mother, the more likely the child was to be in the plant-based cluster. If there were other children in the family, then the study child tended to favor the processed cluster if their siblings were older and the traditional British cluster if the siblings were younger.

Further cluster analysis was performed to assess whether dietary patterns were stable in children longitudinally between the ages of 7 and 13 years.^25^ The 3 sets of food records collected at ages 7, 10, and 13 years were used, and the input variables were average weight (g/d) of each food group consumed. In this analysis, a 4-cluster solution was found to be the most interpretable and most reliable at all 3 ages (<10% reclassified at each age). Table 5 shows the mean weights (g/d) of the foods consumed by the children in the 4 clusters extracted at the age of 7 years.^25^ There were fairly similar numbers in each cluster at each age, and the first 3 clusters were quite similar to those found from the FFQ at age 7 years. The main contributors to the fourth cluster were margarine, sweet spreads, and diet soft drinks. These foods were better measured in the food records than in the FFQ, where they had not been well differentiated; this may be the reason that this cluster was not found in the FFQ analysis.

**Table 5:** Mean (standard deviation) weight (g/d) of foods consumed across clusters for 6837 children at age 7 years with the highest and lowest mean in each row in bold and underlined, respectively

Food item	Processed (n = 1991)	Healthy (n = 1709)	Traditional (n = 1558)	Packed lunch (n = 1579)
Full fat milk	132.3^b^ (174.5)	132.9^b^ (179.7)	**149.6**^a^ (198.3)	80.0^c^ (132.4)
Reduced fat milk	97.6^b^ (143.1)	**143.6**^a^ (178.7)	106.7^b^ (148.5)	132.4^a^ (167.6)
Cheese	6.7^d^ (10.7)	**16.2**^a^ (16.2)	8.5^c^ (11.8)	14.2^b^ (16.5)
Yogurt, fromage frais	28.1^c^ (39.9)	**47.2**^a^ (50.2)	34.4^b^ (43.8)	37.0^b^ (44.9)
Butter, animal fat	2.0^b^ (5.5)	**4.5**^a^ (8.1)	2.4^b^ (5.9)	1.0^c^ (4.3)
Margarine	5.8^c^ (5.2)	6.7^b^ (6.5)	7.2^b^ (6.3)	**15.7**^a^ (7.1)
Vegetable oil	0.1^b^ (0.5)	**0.1**^a^ (0.7)	0.1^b^ (0.4)	0.1^b^ (0.5)
High-fiber bread	5.9^c^ (15.2)	**25.7**^a^ (33.1)	10.1^b^ (20.1)	4.2^c^ (13.9)
Low-fiber bread	43.9^b^ (28.1)	39.6**^c^** (31.8)	45.2^b^ (31.3)	**94.2**^a^ (33.3)
Special bread	1.1^b^ (6.8)	**2.5**^a^ (9.8)	1.0^b^ (5.6)	1.0^b^ (6.2)
Other flour-based products	5.2^b^ (12.9)	7.3**^b^** (16.3)	**9.7**^a^ (16.5)	6.6^b^ (14.6)
Breakfast cereal	29.6^b^ (20.8)	**37.3**^a^ (25.8)	31.4^b^ (22.3)	25.6^c^ (20.7)
Rice	4.1^c^ (12.5)	**9.1**^a^ (20.6)	5.6^b^ (14.8)	4.6^b,c^ (13.7)
Pasta	9.2^c^ (19.5)	**27.0^a^** (32.9)	11.9^b^ (22.6)	13.1^b^ (24.5)
Baked beans, canned pasta	**42.9**^a^ (47.3)	22.6^b,c^ (34.3)	21.2^c^ (29.9)	25.8^b^ (34.4)
Pizza	**12.7**^a^ (25.3)	11.2^a^ (23.6)	6.9^b^ (18.1)	8.8^b^ (21.3)
Eggs	7.3^b^ (15.8)	**9.8^a^** (16.8)	6.4^b^ (13.7)	7.1^b^ (14.7)
Coated and fried chicken	**15.6**^a^ (21.4)	6.8^c^ (14.5)	7.2^c^ (14.7)	9.2^b^ (17.0)
Poultry	11.0^c^ (18.6)	14.9^b^ (21.4)	**25.2**^a^ (27.5)	12.8^c^ (18.9)
Ham, bacon	5.6^c^ (9.6)	7.7^b^ (11.1)	7.9^b^ (11.7)	**10.6**^a^ (14.1)
Red meat	18.6^c^ (27.5)	24.4^b^ (32.0)	**33.7**^a^ (35.9)	22.2^b^ (29.1)
Meat pies, pasties	**6.7**^a^ (17.0)	3.6^c^ (11.0)	6.1^a,b^ (16.4)	5.3^b^ (14.3)
Processed meat	**22.4**^a^ (24.8)	10.0^c^ (15.0)	14.4^b^ (19.8)	14.2^b^ (19.7)
Coated and fried white fish	**11.1**^a^ (17.8)	6.4^b^ (13.5)	6.6^b^ (13.9)	6.8^b^ (14.4)
White fish, shellfish	1.9^b^ (10.2)	**3.1**^a^ (12.6)	2.4^a,b^ (12.6)	1.7^b^ (9.0)
Tuna, oily fish	2.5^b^ (9.8)	**6.2**^a^ (13.8)	3.5^b^ (10.4)	3.4^b^ (10.3)
Vegetarian products	1.4^a^ (11.2)	**4.3**^a^ (23.2)	2.4^b^ (19.5)	1.6^b^ (10.5)
Chips	**52.9**^a^ (32.8)	17.3^d^ (21.6)	20.7^c^ (22.2)	26.2^b^ (25.9)
Roast potatoes	11.5^c^ (19.6)	8.1^d^ (15.7)	**40.9**^a^ (33.0)	14.8^b^ (22.2)
Other potatoes	23.2^c^ (30.7)	33.1^b^ (34.7)	**38.3**^a^ (38.3)	25.0^c^ (30.8)
Root vegetables	1.1^c^ (4.4)	1.8^b^ (5.5)	**3.5**^a^ (8.8)	1.2^c^ (4.4)
Carrots	6.3^d^ (9.9)	11.5^b^ (14.2)	**24.8**^a^ (18.8)	9.0^c^ (11.6)
Green leafy vegetables	3.3^d^ (6.9)	7.1^b^ (11.0)	**17.9**^a^ (17.0)	4.6^c^ (8.7)
Peas, broad beans, sweetcorn	7.8^c^ (12.7)	11.3^b^ (14.8)	**15.4**^a^ (18.1)	8.6^c^ (13.7)
Other cooked vegetable dishes	6.1^b^ (13.7)	11.3^a^ (20.0)	**12.5**^a^ (19.0)	6.5^b^ (13.6)
Salad, tomatoes	7.0^c^ (15.5)	**24.1**^a^ (29.5)	9.8^b^ (18.4)	10.7^b^ (19.1)
Legumes	0.2^c^ (2.0)	**1.1**^a^ (6.7)	0.5^b^ (4.3)	0.4^b^ (4.0)
Soup	4.9^b^ (21.6)	**6.8**^a^ (24.1)	4.8^c^ (19.1)	5.1^b,c^ (20.9)
Nuts, seeds, peanut butter	1.3^b^ (4.8)	**2.7**^a^ (6.9)	1.3^b^ (4.4)	1.4^b^ (4.8)
Fresh fruit	47.5^c^ (54.1)	**121.7**^a^ (84.7)	69.1^b^ (65.3)	67.1^b^ (63.2)
Other fruit	2.7^c^ (11.6)	**6.4**^a^ (17.6)	5.0^b^ (15.7)	3.4^c^ (13.8)
Puddings	10.3^c^ (22.2)	12.5^b^ (24.2)	**17.7**^a^ (27.9)	9.7^c^ (21.0)
Dairy puddings	39.8^b^ (41.7)	35.2^c^ (36.5)	**48.2**^a^ (43.8)	36.6^b,c^ (37.9)
Cakes	23.5^b^ (25.2)	29.1^a^ (29.2)	**29.5**^a^ (28.0)	22.9^b^ (25.5)
Chocolate	**12.6**^a^ (15.9)	8.6^c^ (12.4)	10.1^b^ (12.8)	12.0^a^ (15.4)
Sweets	**8.6**^a^ (12.4)	5.5^c^ (9.1)	6.9^b^ (10.3)	6.4^b,c^ (9.9)
Sugar	**2.9**^a^ (4.9)	1.9^c^ (3.3)	2.7^a,b^ (4.3)	2.5^b^ (4.4)
Sweet spreads	4.2^d^ (7.7)	6.3^b^ (9.3)	5.1^c^ (8.2)	**7.7**^a^ (11.6)
Biscuits	26.8^b^ (20.8)	20.6^d^ (16.8)	22.8^c^ (17.7)	**28.9**^a^ (20.4)
Crackers, crispbreads	1.7^a,b^ (5.1)	**2.1**^a^ (5.2)	1.4^b^ (4.1)	2.0^a^ (5.6)
Crisps	18.0^b^ (13.5)	12.6^d^ (10.9)	16.2^c^ (12.8)	**23.7**^a^ (13.5)
Low energy density sauce	9.3^c^ (11.3)	10.2^c^ (12.0)	**26.4**^a^ (16.9)	12.2^b^ (12.6)
High energy density sauce	0.6^c^ (2.6)	**1.7**^a^ (4.3)	0.8^b,c^ (2.6)	0.9^b^ (2.7)
Salty flavoring	0.2^c^ (0.9)	0.4^b^ (1.1)	0.3^b,c^ (1.0)	**0.6**^a^ (1.6)
Water	99.2^c^ (135.1)	**206.3**^a^ (215.4)	156.5^b^ (187.0)	109.4^c^ (160.8)
Fizzy drinks	**54.7**^a^ (112.1)	29.7^b^ (69.3)	32.4^b^ (76.6)	28.5^b^ (72.3)
Diet fizzy drinks	**123.1**^a^ (164.1)	40.6^d^ (81.7)	82.6^c^ (127.5)	100.7^b^ (145.1)
Squash	**79.1**^a^ (142.3)	67.5^b^ (124.7)	75.5^a,b^ (134.5)	69.9^a,b^ (131.4)
Diet squash	203.1^b^ (222.6)	119.2^d^ (169.5)	177.8^c^ (208.2)	**285.4**^a^ (277.3)
Fruit juice	64.5^c^ (109.4)	**134.6**^a^ (156.4)	76.9^b^ (119.7)	69.9^b,c^ (113.5)
Flavored milk drinks	**18.1**^a^ (49.7)	13.0^b^ (41.2)	13.3^b^ (42.2)	13.3^b^ (44.6)
Tea, coffee	39.8 (90.5)	18.8^b^ (58.0)	37.5^a^ (82.5)	**41.4**^a^ (92.4)

^a^^,^^b^^,^^c^^,^^d^ When values in the same row have a different superscript letter, there is a difference (*P* > 0.05) between cluster means by the Tukey-Cramer method.

Reproduced from Northstone et al. (2012)^25^ with permission.

Exactly the same foods were associated with the processed cluster at ages 7 (Table 5), 10, and 13 years, with the addition of higher full-fat milk and lower soup and cake intake at ages 10 years and 13 years and lower breakfast cereals, puddings, and biscuits intakes at age 13 years only. The “healthy” cluster (Table 5) had many of the characteristics of the plant-based FFQ cluster.^25^ Again, the foods associated were very similar at ages 7, 10, and 13 years; the only difference was a lower intake of full-fat milk at ages 10 years and 13 years compared with the other clusters.

The traditional cluster showed some differences between the age groups in relative amounts of foods consumed. At age 7 years (Table 5), the amount of full-fat milk was higher and the amount of baked beans/canned pasta, pizza, and eggs was lower in this cluster than the others. This was not true at ages 10 years and 13 years, although less coated white fish was eaten in this cluster at the older ages. The 13-year-olds in this cluster drank more tea/coffee and ate more chocolate than those in other clusters. This traditional cluster had many similarities to the traditional British cluster in the FFQ analysis.

The fourth cluster extracted at each age from these data (Table 5) was associated with much higher intakes of white bread and margarine, cold meat, crisps, and dilutable diet drinks; foods that are likely to be part of a “packed lunch” in school-aged children.^25^ At each age, the average intakes of white bread and margarine were more than twice as high in this cluster than the nearest of the other clusters (e.g., 107 g/d white bread and 20 g/d margarine in the packed lunch cluster compared with 51 g/d and 8 g/d, respectively, in the traditional cluster at age 10 years). Sweet and savory spreads (salty flavorings) were also higher, as were biscuits; however, butter and some types of fish were lower. At the 2 younger ages, slightly more tea/coffee was drunk by the children in this cluster than by those in the other clusters. A similar packed lunch pattern had been found when PCA was applied to the food records collected at age 10 years but not in the FFQ PCA or cluster analysis in childhood.

**Figure 1:**
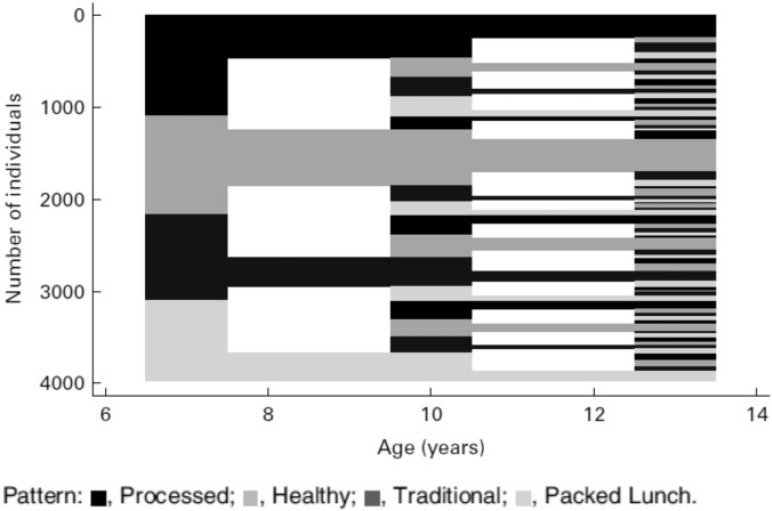
Sequence index plot illustrating changes in cluster membership over time. Reproduced from Northstone et al. (2012)^25^ with permission

The stability of the clusters over the 3 food records kept for the same children at ages 7, 10, and 13 years was examined (n = 4405–5287).^25^ The healthy cluster was the most stable, with half of the children in that cluster at age 7 years remaining in it at later ages (see Figure 1). The processed cluster was the next most stable, with approximately 40% remaining in that cluster across the ages, whereas the other 2 clusters retained a quarter to a third of the children each time (Figure 1). Higher maternal educational attainment was the strongest determinant of whether a child was in the healthy diet cluster at ≥2 time points (compared with children whose mothers had the lowest educational attainment, those with highly educated mothers were 4.39 times [95% CI, 3.05–6.35] more likely to be in the healthy cluster at least twice). Children of older mothers rather than younger mothers were also more likely to remain in the healthy cluster, as were girls rather than boys. These data suggest that children introduced to either a healthy or a processed type of dietary pattern by 7 years of age are likely to continue with this type of pattern into adolescence.

The cluster patterns provide some useful insights into what a healthy diet might look like in the context of children growing up in the United Kingdom in the early 21^st^ century, particularly in contrast to a processed diet.^25^ At each age, the children in the healthy cluster ate, on average, more than double the amount of fruit (122 g/d at 7 y) than the children in the processed cluster (47 g/d at 7 y), but these amounts did not increase as the children grew older. Similarly, salad vegetables were higher in the healthy cluster (24 g/d at 7 y) than in the processed cluster (7 g/d at 7 y); in this case, intakes in both clusters increased with age to 42 and 14 g/d, respectively, at age 13 years. Crisps were eaten in smaller amounts by the healthy cluster children (13 g/d at 10 y) than the packed lunch cluster children (24 g/d at 10 y). A change in the type of milk consumed occurred in all of the clusters as the children grew older; at age 7 years, amounts consumed were slightly in favor of full-fat milk, but by age 13 years, low-fat milk intake was more than double that of full-fat milk in all clusters. Overall, milk intake was lower at age 13 years than at age 7 years, and the processed cluster children had the lowest intake (157 g/d at 13 y), whereas the healthy cluster children had the highest intake (202 g/d at 13 y). Cross-sectional preliminary analysis of the average nutrient profiles of the cluster patterns showed almost identical relationships at each age. In general, the data suggest that the healthy and traditional patterns have better micronutrient profiles than the processed and packed lunch patterns.

### Dietary patterns from food records in childhood using reduced rank regression

An analysis using RRR was carried out in the Children in Focus subsample of ALSPAC children using food records collected at ages 5 years and 7 years (n = 521 at 5 y and n = 682 at 7 y) and fat mass measured by DXA at age 9 years.^26^ The amount of likely misreporting of EI was determined: 72% of children at age 5 years and 73% at age 7 years had plausible reports of EI. The prevalence of overweight was significantly higher among under-reporters; therefore, the analysis included a categorical variable accounting for misreporting status. The “energy-dense, high-fat, low-fiber” dietary pattern obtained was highly correlated with dietary energy density (*r* = 0.81) and fiber density (*r* = −0.73) and moderately correlated with percentage of energy from fat (*r* = 0.49) at age 7 years, with very similar correlations at age 5 years. The foods with the highest factor loadings were similar at each age. The pattern scores tracked strongly between ages 5 years and 7 years (intraclass correlation: 0.66 [95% CI, 0.60–0.71]) suggesting that, for the most part, children with high scores at age 5 years were likely to have high scores at age 7 years. Children whose mothers were in the lowest education group and those who watched television for >2 hours/day (from a questionnaire when the child was 4.5 y old) were more likely to have high scores on the energy-dense, high-fat, low-fiber diet at ages 5 years and 7 years.^26^

There were no associations between quintiles of pattern score at age 5 years or age 7 years and mean BMI or overweight (defined using age- and sex-specific BMI cut-offs from the International Obesity Task force) at ages 5, 7, or 9 years.^26^ However, when FMI was used to assess adiposity at age 9 years, there were associations with the dietary pattern score quintiles at age 7 years but not those at age 5 years. Mean FMI was 1.03 (95% CI, 0.70–1.42) in the lowest quintile and 1.17 (95% CI, 0.80–1.61) in the highest quintile of pattern score (*P = *0.04), and excess adiposity increased from 13% to 23% (*P = *0.01) between these quintiles. In the multivariate model (adjusted for sex, height at 9 y, misreporting status, maternal BMI, maternal education [5 categories], baseline BMI of the child, and child’s hours of television watching) a 1 standard deviation increase in pattern score was associated with an increase in fat mass of 0.28 kg (95% CI, 0.05–0.53; *P = *0.02). This was only slightly attenuated by controlling for EI. There was a positive linear trend between the adjusted odds of excess adiposity at age 9 years and quintiles of dietary pattern score at age 5 years (*P = *0.03) and age 7 years (*P < *0.0001). A child in quintile 5 compared with quintile 1 of the dietary pattern score at age 5 years was 2.52 (95% CI, 1.13–6.08) times more likely to have excess adiposity at age 9 years, and a child in quintile 5 compared with quintile 1 of the dietary pattern score at age 7 years was 4.18 (95% CI, 2.07–9.38) times more likely to have excess adiposity at age 9 years.

**Figure 2:**
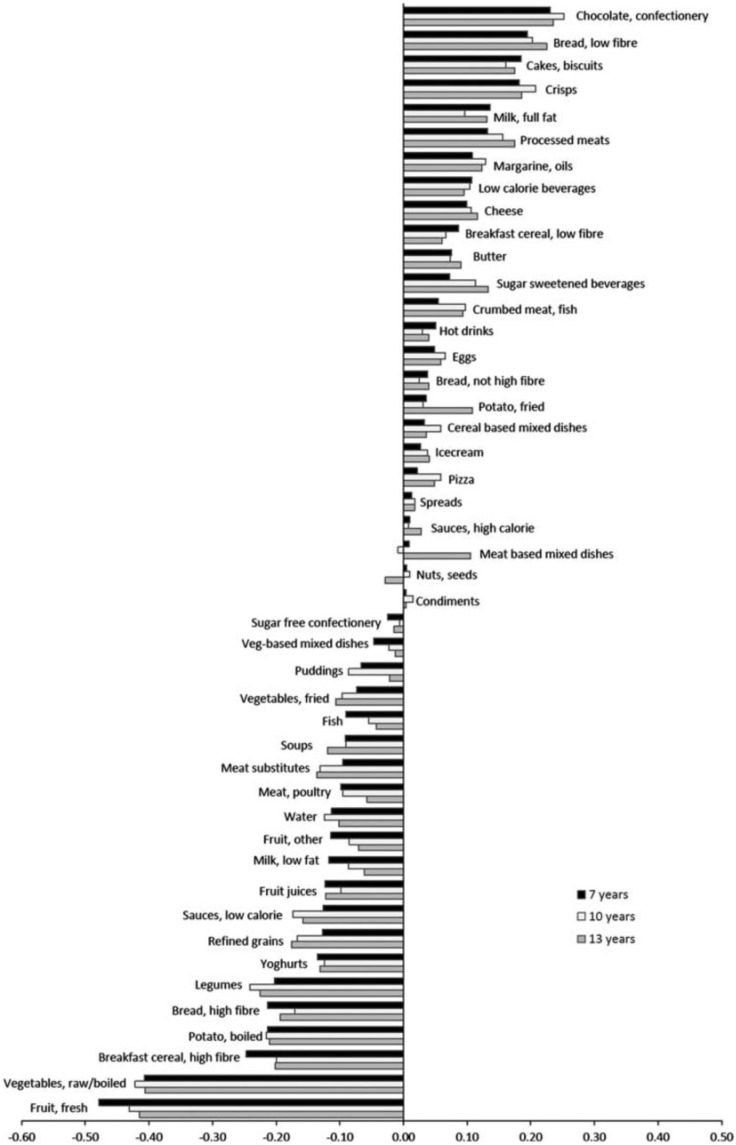
Food group factor loadings from reduced rank regression analysis for the “energy-dense, high-fat, low-fiber” dietary pattern at ages 7, 10, and 13 years using food records from the ALSPAC cohort. Reproduced from Ambrosini et al. (2012)^27^ with permission.

The RRR analysis was extended to cover the whole cohort using the food records at ages 7, 10, and 13 years, and the relationship of the resulting dietary patterns with fat mass at ages 11, 13, and 15 years (n = 6772) was assessed.^27^ The factor loadings for the food groups on the energy-dense, high-fat, low-fiber dietary patterns at the 3 ages are shown in Figure 2. At age 7 years, >80% of the variation in the dietary pattern was explained by the top 4 and bottom 6 food factor loadings (see Figure 2). This was similar at the other 2 ages. Each child received a dietary pattern *z*-score representing the degree to which their reported dietary intake reflected this dietary pattern at each age. Linear regression analysis between dietary pattern *z*-score and FMI *z*-score was performed. Fully adjusted dietary pattern *z*-scores at ages 7 years and 10 years were positively associated with later fat mass; however, at age 13 years, full adjustment attenuated the association to the null (see Table 6). Most of the attenuation at all ages occurred when maternal education and prepregnancy BMI were included in the adjustment. These results suggest that the relationship between the dietary pattern and FMI becomes weaker with age.

**Table 6:** Prospective associations between an energy-dense, high-fat, low-fiber dietary pattern (*z*-score), and fat mass index (*z*-score)

Characteristic	FMI *z*-score^a^	FMI *z*-score^a^	FMI *z*-score^a^
Age (y)	11	13	15
Dietary pattern at 7 y	n = 4002	n = 3472	n = 2626
β (95% CI), *P*-value	0.07 (0.05–0.10), <0.001	0.06 (0.03–0.09), <0.001	0.06 (0.03–0.10), <0.001
Dietary pattern at 10 y	n = 4571	n = 3943	n = 3425
β (95% CI), *P*-value	0.03 (0.01–0.05), <0.005	0.04 (0.01–0.06), <0.001	0.03 (0.01–0.06), <0.001
Dietary pattern at 13 y			n = 3005
β (95% CI), *P*-value			0.01 (−0.01 to 0.03), 0.348

^a^Linear regression adjusted for child’s sex, age at dietary assessment, dietary misreporting, physical activity at 11 years, maternal education, and prepregnancy body mass index.

*Abbreviations*: CI, confidence interval; FMI, fat mass index.

Modified from Ambrosini et al. (2012)^27^ with permission.

The degree of tracking of this energy-dense, high-fat, low-fiber dietary pattern over the 3 ages was assessed using applied dietary pattern scores based on the pattern scores at age 7 years.^28^ A tracking coefficient for the dietary pattern was calculated such that a coefficient of 1 indicated perfect tracking and a coefficient of 0 indicated no tracking at all. Tracking coefficients for all food groups were calculated in the same way (see Table 7). The overall pattern score tracked more strongly than the individual foods. Furthermore, the foods negatively associated with the dietary pattern tracked more strongly than the positively associated foods (Table 7). This suggests that children who have low intakes of these foods by the time they are aged 7 years are likely to continue this pattern.^28^

**Table 7:** Tracking coefficients for the energy-dense, high-fat, low-fiber dietary pattern and food group *z*-scores from 7 to 13 years of age

Dietary pattern and food	Boys	Girls
	Tracking	Tracking
	coefficient^a^	coefficient^a^
	(95% CI)	(95% CI)
Energy-dense, high-fat, low-fiber dietary pattern	0.48	0.38
	(0.44–0.52)	(0.35–0.41)
Food groups positively loaded onto the dietary pattern		
Confectionery, chocolate	0.18	0.14
	(0.14–0.21)	(0.11–0.17)
Bread, low-fiber	0.22	0.17
	(0.19–0.25)	(0.14–0.20)
Cakes, biscuits	0.22	0.15
	(0.19–0.25)	(0.12–0.18)
Potato crisps	0.23	0.23
	(0.20–0.26)	(0.20–0.25)
Milk, full-fat	0.40	0.31
	(0.36–0.45)	(0.27–0.34)
Processed meat	0.15	0.14
	(0.12–0.18)	(0.11–0.18)
Food groups negatively loaded onto the dietary pattern		
Fresh fruit	0.35	0.31
	(0.32–0.39)	(0.28–0.35)
Vegetables, raw or cooked	0.31	0.28
	(0.28–0.35)	(0.25–0.31)
Breakfast cereal, high-fiber	0.21	0.14
	(0.17–0.26)	(0.10–0.17)
Potato, boiled	0.15	0.14
	(0.11–0.19)	(0.10–0.18)
Bread, high-fiber	0.23	0.22
	(0.20–0.27)	(0.19–0.26)
Legumes	0.21	0.15
	(0.16–0.26)	(0.11–0.18)

Reproduced from Ambrosini et al. (2014)^28^ with permission.

Maternal factors were investigated in analyses adjusted for misreporting status.^28^ Decreasing maternal educational attainment was associated with increasing dietary pattern *z*-score and increasing energy-adjusted food intake *z*-score of some food groups, such as chocolate/confectionery, crisps, and processed meat. There were associations with decreasing intake of other foods, such as fresh fruit, vegetables, high-fiber breakfast cereals, brown/wholemeal bread, and cakes/biscuits. The relationship of the dietary pattern scores and food intakes with increasing maternal prepregnancy BMI showed fairly similar associations, i.e., positive with dietary pattern *z*-score, white bread, and processed meat and negative with cakes/biscuits, fresh fruit, and vegetables. These finding suggest that these maternal characteristics are associated with higher scores on a dietary pattern that is related to increased adiposity in children and adolescents.

### Child’s educational attainment, cognitive development, and behavioral outcomes associated with dietary patterns

The educational attainment of ALSPAC children was assessed nationally at school entry (4–5 y), at key stage 1 (6–7 y) and key stage 2 (10–11 y). Associations with PCA-generated dietary patterns from the FFQs at ages 3, 4, and 7 years were examined (n = 4244).^29^ High scores on the junk pattern at age 3 years were consistently related to lower educational attainment at all 3 stages of assessment. The other patterns at age 3 years and all patterns at other ages were not consistently associated with level of educational attainment. However, the β coefficient of the association of the junk pattern at age 3 years with key stage 2 scores was very small (β = −0.027 [SE 0.014]; *P < *0.05) compared with that with prior attainment scores (β = 0.687 [SE 0.013]; *P < *0.01), suggesting a small but independent role for preschool diet. These results were adjusted for child and maternal factors, including breastfeeding and maternal educational attainment.

IQ was measured in the children at age 8 years and assessed in relation to the PCA dietary pattern scores from ages 3 years to 9 years (n = 3966 with complete data).^30^ In the fully adjusted model, which took account of the sex and age (at testing) of the child, breastfeeding duration, maternal age and educational attainment, housing tenure, measures of parenting, and all other dietary pattern scores, IQ was negatively associated with the junk pattern score at age 3 years (β = −1.67 IQ points; 95% CI, −2.34 to −1.00; *P < *0.0001) and positively associated with the health-conscious/vegetarian pattern score at age 9 years, close to the age of IQ assessment (β = 1.20; 95% CI, 0.52–1.88; *P = *0.001). There were no adjusted associations with dietary pattern scores at age 4 years or age 7 years. These results are consistent with the child’s educational attainment data (above), which used entirely different methods of assessment applied nationally to all children in state schools. Taken together, the results suggest early diet (preschool) is more important in relation to cognitive development than later diet. This may be linked to the lower intake of micronutrients associated with higher scores on the processed types of dietary patterns.^20^

In the children, behavioral problems had been assessed by the SDQ at ages 4 years and 7 years, and scores for hyperactivity, conduct problems, emotional problems, peer problems, and prosocial behavior were obtained.^31^ Children with behavioral problems incident between ages 4 years and 7 years were identified and compared with problem-free children (n = 4430). Children with high frequency of behavioral problems before age 4 years were excluded. Incident problems were assessed in relation to the processed dietary pattern scores obtained from PCA using the FFQ when the child was aged 4 years. The only incident behavioral problem consistently associated with the dietary pattern was hyperactivity (odds ratio for hyperactivity if the child was in the highest compared with the lowest tertile for processed dietary pattern score: 1.16; 95% CI, 1.07–1.27; after adjustment for child and maternal factors). Further adjustment for the IQ of the child in a subset of the children did not attenuate this association greatly.^31^ A further analysis used the SDQ assessments at ages 7 years and 8 years and investigated incident behavioral problems in relation to the processed dietary pattern consumed by the children at age 7 years (n = 4220).^32^ The few associations found in the univariate analysis were completely abolished on controlling for the same factors as above. This analysis also looked separately at added sugar intake and found no adjusted associations. A long-term effect of this type of diet in mid-childhood on behavior problems is not well supported by these data.

Box 1 Key findings in brief

- Similar dietary patterns were found in a cohort of children at various ages between 3 years and 13 years and in their mothers and fathers using principal components analysis
- The health-conscious and traditional patterns of diet extracted at all ages were associated with better nutrient profiles than the processed patterns, which tended to be energy-dense and nutrient-poor
- Dietary patterns from cluster analysis grouped the children by foods recorded at ages 7, 10, and 13 years; many of the children continued with the same dietary pattern at each age, with the health-conscious and processed patterns being the most stable
- An “energy-dense, high-fat, low-fiber” dietary pattern was extracted from food recordings at ages 7, 10, and 13 years using reduced rank regression analysis. High scores on this pattern were associated with increasing adiposity between 11 and 15 years in these children
- A major determinant of the dietary patterns extracted from all 3 methods was the amount of fruits and vegetables consumed; the healthier diet patterns contained more fruits and vegetables, whole-grain cereal products, and plain potatoes; the less healthy patterns contained more chocolates/confectionery, white bread, cakes/biscuits, and crisps
- Mother’s educational attainment was a strong determinant of the dietary patterns extracted using all 3 methods; higher educational attainment was associated with healthier, more nutrient-dense dietary patterns

### Maternal influences on dietary patterns in childhood

Dietary patterns from PCA using the FFQs in childhood (ages 3–9 y) were investigated in relation to the presence of eating disorders in the mothers to assess whether children of those with eating disorders have a different type of diet from children whose mothers are not affected.^33^ During pregnancy, the women with eating disorders had been shown to be more likely not to eat meat (odds ratio, 2.8; 95% CI, 2.1–3.8; *P < *0.001) and to have higher average scores on a vegetarian dietary pattern (β = 0.3; 95% CI, 0.2–0.4; *P < *0.001) than women without such disorders.^69^ Children (n = 9423) were divided according to their mother’s history of eating disorders before or during the study pregnancy: anorexia nervosa (n = 140, 1.4%), bulimia nervosa (n = 175, 1.9%), and a combination of the 2 occurring in the same person (n = 71, 0.8%). Children of anorexics and bulimics but not the combined diseases had higher scores on the health-conscious patterns at ages 3, 4, and 7 years and on the health-conscious/vegetarian pattern, which only occurred at age 9 years, than children of women without these disorders. There was some evidence that this was less likely in boys than in girls of anorexic mothers. There were no differences between the groups in the processed pattern scores; however, there was evidence that, overall, the average processed pattern score in children increased with age. In the younger children, all 3 exposed groups had lower average scores on the traditional patterns compared with the unexposed group. However, these average scores increased with age in the 3 exposed groups and were very similar to, if not higher than, those in the unexposed group by age 9 years. Overall, the data suggest that mothers with eating disorders do feed their children differently from mothers without eating disorders but that these differences become less marked as children grow older, perhaps due to greater outside influences.

## DISCUSSION

Similar patterns of eating emerge both in children at each age and in their parents; these have some common features regardless of which method of obtaining dietary patterns is used. Higher consumption of fruit and vegetables, wholegrain bread and breakfast cereal, plain potatoes, and low-fat milk are always associated with better scores on the healthier types of patterns,^12^^,^^15^^,^^24^^,^^27^ which tend to have better nutrient profiles.^19^^,^^20^ These foods usually have lower scores on less healthy types of patterns, which tend to have poorer nutrient profiles.^19^^,^^20^ The less healthy, processed patterns usually include higher consumption of white bread, low-fiber breakfast cereals, fried potatoes (chips), full-fat milk, chocolate/confectionery, and crisps.^12^^,^^15^^,^^24^^,^^27^ The processed patterns are often associated with poorer outcomes—e.g., high scores on the junk pattern identified at age 3 years were associated with a poorer nutrient profile^13^ and worse cognitive development in mid-childhood.^29^^,^^30^

The different sources of data (FFQ or food record) provide some different insights into diets consumed during childhood. The food records covered in detail all foods that were consumed over a short period of time, so new patterns, such as the packed lunch pattern, which did not occur in the FFQ analyses,^24^ were derived from both the PCA^23^ and cluster analysis^25^ using the food records. Thus, the availability of different types of food intake data enhanced the degree of understanding of the diet that could be achieved. The other 3 patterns obtained were very similar between the FFQ and food record, as was the case in other studies that have compared the results of PCA using FFQ and food records in the same subjects.^78^^,^^79^ Where the PCA and cluster analysis patterns were directly compared using the FFQ at age 7 years,^24^ the agreement between the methods was very similar to that found in the Southampton Women’s Survey comparing patterns derived by both methods in nonpregnant women.^7^ In the Southampton Women’s Survey, the prudent diet PCA score was strongly positively related to the healthy cluster, very similar to the relationship between the health-conscious PCA score and the plant-based cluster in ALSPAC children.

There was evidence of tracking of the dietary patterns using all 3 methods of deriving dietary patterns and using both FFQs^16^ and food records.^25^^,^^26^^,^^28^ Particular insight was gained from the cluster analysis, which, by placing the children into distinct groups, allowed a direct comparison of the same pattern allocated at each age, thus showing that the healthy pattern tracked most strongly, followed by the processed pattern (Figure 2).^25^ This was confirmed in the RRR, where foods associated negatively with the energy-dense, high-fat, low-fiber dietary pattern tracked more strongly than the foods associated positively with the pattern.^28^ Both the cluster analysis and the RRR showed that the dietary pattern of the child at age 7 years was a strong determinant of the dietary pattern of the child at ages 10 years and 13 years.

The development of obesity was assessed using the patterns derived from both PCA^23^ and RRR.^26^^,^^27^ Strong associations were found between increasing scores on the RRR-derived energy-dense, high-fat, low-fiber dietary pattern from ages 7 years to 10 years to 13 years and increasing obesity/adiposity up to age 15 years, measured by FMI.^27^ The response variables used had previously been shown to be related to obesity in adults. There was evidence that the pattern score at age 7 years was more strongly predictive of adiposity at age 15 years than scores at later ages.^27^ The RRR-derived pattern has many features of the processed patterns identified by the other dietary patterns methods. In particular, low intakes of fruits and vegetables were strongly negatively associated with this pattern (Figure 1). The same pattern derived from food records collected at ages 5 years and 7 years was shown to be related to adiposity but not to BMI at age 9 years.^26^^,^^68^ At age 10 years, the PCA-derived health-aware pattern characterized by higher consumption of fruit and vegetables, brown/wholemeal bread, pasta, cheese, and fish and lower consumption of chips, crisps, processed meat, and carbonated drinks was associated with lower fat mass at age 11 years.^23^

These data suggest that action to increase young children’s intake and liking of fruits and vegetables, as well as whole-grain products, is likely to provide progress in tackling the obesity epidemic and that this action should start before the age of 7 years to be most effective.

Insight into obesity development in children participating in the ALSPAC benefited from the fact that fat and lean mass had been measured using DXA at several ages. This is a much more sensitive method for identifying problematic body composition than the use of BMI definitions of obesity alone.^72^^,^^80^ Furthermore, use of a longitudinal design in some of the studies has allowed the gains in fat mass and lean mass associated with the dietary patterns to be assessed,^27^ which is a more meaningful tool than using current body composition at each age.

Using all 3 methods, the patterns obtained were highly influenced by the characteristics of the child’s mother, particularly her educational attainment, age at the birth of the child, and prepregnancy BMI. Higher maternal educational attainment is associated with high scores on the healthier patterns,^15^^,^^17^^,^^23^^,^^25^ and lower maternal educational attainment, age, and higher prepregnancy BMI are associated with higher scores on the less healthy patterns.^15^^,^^25^^,^^28^ Family characteristics, such as the number of older or younger siblings in the family, also influence the types of foods consumed by the child.^13^^,^^15^^,^^17^^,^^18^^,^^24^ The child’s own characteristics, such as being choosy with food, are related to the dietary pattern scores, being associated with lower scores on the healthier patterns and higher scores on the less healthy patterns in the PCA analysis.^17^^,^^18^ These insights into the social patterning of diet may help to explain the social patterning of health outcomes.^81^

Dietary patterns are associated with the nutrient content of the diet. Higher scores on both the healthy and traditional types of patterns are associated with higher micronutrient content of the diet in both adults and children.^19^^,^^20^ In the opposite direction, higher scores on the processed pattern types are associated with higher energy and lower micronutrient content.^19^^,^^20^ The data suggest that these processed pattern types can be described as “energy-dense, nutrient-poor” ways of eating.

The PCA using the FFQ derived a vegetarian or semi-vegetarian type of pattern in the adults (both women and men),^10–12^ and this pattern was found in the diets of the children at older ages.^16^^,^^17^ There was evidence of it as part of the health-conscious pattern at age 9 years,^16^ with a recognizably vegetarian pattern described at age 13 years.^17^ This pattern did not have a strong relationship with nutrient intakes in either adults or children.^19^^,^^20^ High scores were associated with the person reporting being a vegetarian (approximately 5.4% of women, 3.0% of men, and 3.5% of 9-year-olds). There had been some suggestion of an association between high scores on this pattern in mothers during pregnancy and high frequency of anxiety symptoms at the same time^34^. Possible associations of this pattern with anxiety or other similar symptoms in the men or the adolescents has not yet been investigated. There was no evidence of a vegetarian type of pattern being extracted from the food record analysis in the children or adolescents either by PCA or cluster analysis, probably because a 3-day food record does not provide enough information for this type of diet to become apparent.

Although often living in the same family home, male and female partners had slightly different dietary patterns,^12^ confirming it is advisable to look at men and women separately when studying diet. There was evidence of differences in dietary pattern scores between males and females as early as mid-childhood.^13^^,^^15^^,^^17^^,^^23^^,^^25^^,^^28^

From the analyses relating dietary patterns in the children to behavioral and cognitive outcomes, the association of high scores on the junk/processed pattern in preschool children with lower educational attainment^29^ and IQ^30^ in later childhood were robust to adjustment, but dietary pattern associations with behavioral problems were not.^31^^,^^32^ This could be due to the associations of high scores on the processed patterns with less favorable measures of SEB,^13^^,^^15^ which are, in turn, associated with worse behavioral and cognitive outcomes, thus possibly confounding the associations.

Some methodological issues associated with dietary patterns analysis have been explored, and these articles have added to the sparse literature in this field. In particular, the investigation into the best stage at which to apply energy adjustment in PCA analyses showed that there is no benefit from adjusting the food group intakes prior to performing the PCA.^21^ Careful assessment of the effect of different input variables from food records on the PCA patterns showed that adjusting each food group variable for total energy intake prior to running the PCA has no advantage over using the average weight of each food group consumed unadjusted.^22^ However, using binary yes/no variables in a PCA may provide valuable insights, particularly in relation to food choices.^22^

The main strength of the ALSPAC is the derivation of dietary patterns at many ages during the childhood of a cohort of children and in their parents, as well as the use of 3 different methods to derive the patterns. Additional strengths are the relatively large sample size, adjustment for confounders, and, in some analyses, physical activity measurement,^23^^,^^27^ although the possibility of residual confounding cannot be ruled out. The numbers of subjects involved in these studies are substantially larger than in many of the studies that have been performed previously. Adjustment for physical activity was made using accelerometer data as an objective measurement rather than self-report; however, like dietary measurements, this method may be susceptible to invalid reporting. As with all longitudinal studies of free-living populations, there was loss to follow-up, and it could be argued that any changes seen in overall dietary pattern scores over time were a result of biases in those who were followed up (Table 2). However, the differences in mean pattern scores are small between time points, particularly those that are close together; therefore, the authors contend that the changes shown are real and not unduly affected by such bias.

PCA and cluster analysis have faced criticism because they are data dependent and the patterns obtained can, therefore, not be directly compared across studies, countries, and cultures. Furthermore, in PCA a number of subjective decisions have to be made during the analytical process,^82^^,^^83^ and cluster analysis can be very sensitive to outliers.^6^ These 2 methods can give results that complement each other; in these analyses, there were some features that cluster analysis identified but PCA did not, particularly in relation to milk and bread consumption. PCA provides a continuous variable for each subject, and this may be preferable in some types of analyses. However, if using a categorical variable would be more meaningful, cluster analysis may be a better choice.^67^ RRR relies on identifying, in advance, variables that are likely to be related to the outcome of interest. In this case, studies of adults had identified such variables in relation to obesity; however, this may not be possible for many other important outcomes.

## CONCLUSION

Uniquely in the ALSPAC, dietary patterns were derived using 3 different methods to evaluate the data collected at different ages throughout childhood and in parents in the same cohort. This has led to many insights into how foods are consumed together in a highly developed country in which food stuffs are readily available and relatively cheap. All 3 methods used to derive dietary patterns confirm that fruit, vegetable, and whole-grain intakes define whether a person is likely to be consuming a healthy diet or not. This suggests that public health initiatives that aim to improve fruit, vegetable, and whole-grain intake as part of a balanced diet are important. It is also clear that the differences in dietary patterns are strongly related to social inequality, particularly with regard to the educational attainment of the mother. These differences may well be contributing to the large inequalities in health outcomes seen in the United Kingdom.^81^
